# The Next Frontier: Unveiling Novel Approaches for Combating Multidrug-Resistant Bacteria

**DOI:** 10.1007/s11095-025-03871-x

**Published:** 2025-06-16

**Authors:** Praveen Mallari, Leila D. Rostami, Ida Alanko, Fadak Howaili, Meixin Ran, Kuldeep K. Bansal, Jessica M. Rosenholm, Outi M. H. Salo-Ahen

**Affiliations:** 1 Department of Zoology, Indira Gandhi National Tribal University, Amarkantak, Madhya Pradesh 484887 India; 2 Pharmaceutical Sciences Laboratory, Faculty of Science and Engineering, Pharmacy, Åbo Akademi University, 20520 Turku, Finland; 3 Structural Bioinformatics Laboratory, Faculty of Science and Engineering, Biochemistry, Åbo Akademi University, 20520 Turku, Finland; 4 Turku Bioscience Centre, University of Turku and Åbo Akademi University, Turku, Finland

**Keywords:** Alternative drug discovery strategies, Antibacterial development, Antibiotic resistance, Next-generation antibiotics, Novel drug delivery mechanisms

## Abstract

**Background:**

The rapid occurrence of bacterial antibiotic resistance poses a significant threat to public health worldwide. Since particularly multidrug-resistant (MDR) pathogens are becoming untreatable with currently available antibiotics, new treatment modalities must be deployed.

**Objectives:**

This review explores the recent advancements and the enduring challenges in new antibacterial development for drug-resistant organisms.

**Results:**

We describe how bacterial resistance to antibiotics arises and discuss why the traditional drug discovery routes are inefficient. The best alternative strategies to overcome these challenges might include exploring new bacterial pathways, utilizing compounds with antibacterial activities from the human microbiome, and repurposing existing drugs. Moreover, novel drug delivery mechanisms that leverage, for example, nanotechnology-based carriers may be breakthrough ideas that can increase antibiotic efficacy and, at the same time, reduce toxicity. Current clinical trials of next-generation drugs indicate that some treatments possess excellent potential to overcome the MDR issue.

**Conclusion:**

Despite the substantial obstacles to getting bench findings to the patient, numerous scientists are still working towards this goal. Both the application of antibiotic stewardship principles and timely considerations through the regulatory pathways are needed to release the next generation of antibiotics that are suitable for the fight against superbugs.

## Introduction

### Emergence of Multidrug-Resistant Bacteria

Over the past century, the discovery of antibiotics has revolutionized medicine, enabling us to swiftly address infections that were once terrifying and mostly fatal. However, bacterial resistance to antibiotics, caused by excessive usage and misuse of these life-saving drugs over the last few decades, has led us to a significant public health crisis that develops and spreads at an alarming rate [1–3]. Namely, bacteria can rapidly evolve antibiotic resistance mechanisms and resist previously effective drugs. The growth of multidrug-resistant organisms (MDROs), resistant to multiple antibiotics, is widespread and must be paid attention to [4]. Multidrug-resistant (MDR) infections pose significant challenges because they cannot be managed with current antibiotic treatments. This leads to longer illnesses, increased mortality rates, and extended hospital stays that incur higher medical costs [5].

In the USA alone, nearly 3 million microbial infections annually are reported along with antibiotic resistance, with a mortality rate of 35,000 deaths [6]. In 2020, according to the report by the European Centre for Disease Prevention and Control (ECDC), nearly 100 people died every day from bacterial infections that are resistant to antibiotics within the European Union (EU)/European Economic Area (EEA) [7]. The number of people infected by antibiotic-resistant bacteria in Europe was over 800,000 that same year and ca. 70% of the infections were healthcare-associated. The situation with the spread of antibiotic-resistant bacteria and the resulting infections and deaths worsened between 2016 and 2020 in the EU countries, leading to increased healthcare costs. According to the most recent ECDC report (data from 2023) [8], the AMR levels in EU/EEA still remain high. In 2019, roughly 1.27 million deaths were directly attributed to bacterial antimicrobial resistance (AMR) globally, according to a study published in the Lancet [9]. Furthermore, the same study identified that approximately 5 million deaths were somehow associated with bacterial AMR that year worldwide. Without introducing alternative anti-MDRO strategies, the mortality rate could rise to 10 million people worldwide per year by 2050, as stated by one of the projections [10]. That would also have a substantial economic impact on the global economy [11].

Resistance to first-line antibiotics and the appearance of bacteria resistant to multiple antibiotics limits the treatment options for doctors and pharmacists [12]. The increasing problem of antibiotic resistance undermines our ability to conduct medical procedures such as a simple diagnosis or a complex surgical operation [13]. Without well-working antibiotics, operations such as hip replacements, caesarean sections, and chemotherapy would involve a high risk of infection [1]. This will pave the way for a future when cancer will no longer be the number one death cause, but infectious diseases have taken over.

**Fig. 1:**
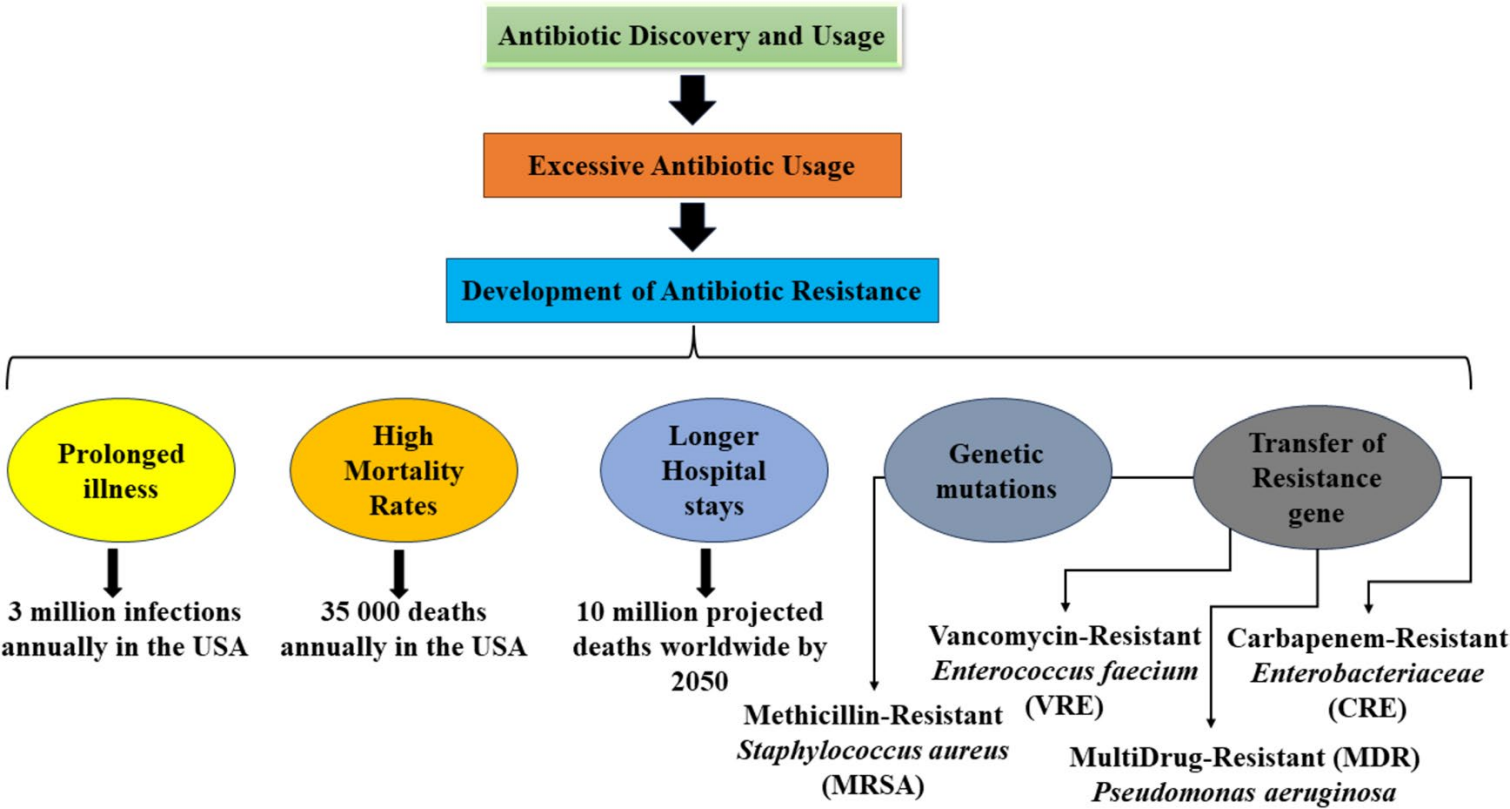
Emergence of multidrug-resistant bacteria.

Most of the antibiotics in current use (including streptomycin, tetracycline, chloramphenicol, erythromycin and vancomycin) can be identified and isolated from the metabolites of the Actinomycetes – a group of gram-positive bacteria [14]. Actinomycetes produce these metabolites to inhibit other microorganisms in their natural habitat. Thus, these organisms are naturally adapted (resistant) to their metabolites. The continuous development of resistance by bacteria to various antibiotic agents is an ancient phenomenon. Some studies document fully functioning resistance genes in microorganisms embedded in permafrost that existed thousands of years ago [15, 16]. Bacteria can develop antibiotic resistance via genetic mutations or through the vertical or horizontal transfer of resistance genes to other strains within and outside the species [15]. The overuse and misuse of antibiotics further speed up the phenomenon as the bacteria can evade the effects of these drugs by applying selection pressure on them and allowing resistance genes to spread and enhance the resistance of the microbial communities in Fig. 1. The most alarming is the diffusion of multidrug efflux pumps and carbapenemase enzymes that provide Gram-negative pathogens like *Escherichia coli* (*E. coli*) and *Klebsiella pneumoniae* (*K. pneumoniae*) to become resistant against nearly all recommended antibiotics [17, 18].

MDROs (so-called “superbugs”) that were previously transmitted mainly in the hospital are now being acquired also in community-associated settings and are spreading more in the community [19]. The most typical MDROs that are being considered severe threats in the healthcare setting include methicillin-resistant *Staphylococcus aureus* (MRSA), vancomycin-resistant *enterococci* (VRE), multidrug-resistant *Pseudomonas aeruginosa* (*P. aeruginosa*) and carbapenem-resistant Enterobacteriaceae (CRE). MRSA is a Gram-positive bacterium resistant to nearly all beta-lactam antibiotics [20]. Multidrug-resistant Gram-negative CRE is, on the other hand, the leading cause of the deaths of around 50% of superbug-infected patients [21]. Moreover, *K. pneumoniae*, *Acinetobacter baumannii* (*A. baumannii*) and Enterobacteriaceae are commonly associated with healthcare-associated infections (HAIs) and are increasingly becoming resistant to most antibiotics, making previously effective drugs less effective in managing such infections [22, 23, 39].

The rapid spread of MDROs highlights the necessity for coordinated actions worldwide to fully scale up policies to use those existing antibiotics more sparingly, develop new antibiotics with new modes of action, and curtail the emergence of such resistant strains. Measures must be undertaken instantly to renew the antibiotic pipeline. New drug discovery methodologies must be developed in designing vaccines and small molecular drugs to combat bacterial resistance [24].

### Urgent Need for Novel Antibiotic Strategies

Although the resistance keeps growing [25], the speed at which new antibiotic compounds are developed and approved for use in clinical settings has decelerated dramatically. The pipeline of new antibacterial drug development remains too inadequate to cope with this dangerous trend of MDR. Over two decades, from 1980 until 2000, more than 20 new antibiotic classes were introduced. But, for this century, just three new compound classes have made it to the drug market. Besides this, economic factors play an essential role in drug development. Unlike antibiotics, drug discovery and development for treating chronic diseases is commercially viable for pharmaceutical companies [26].

**Fig. 2:**
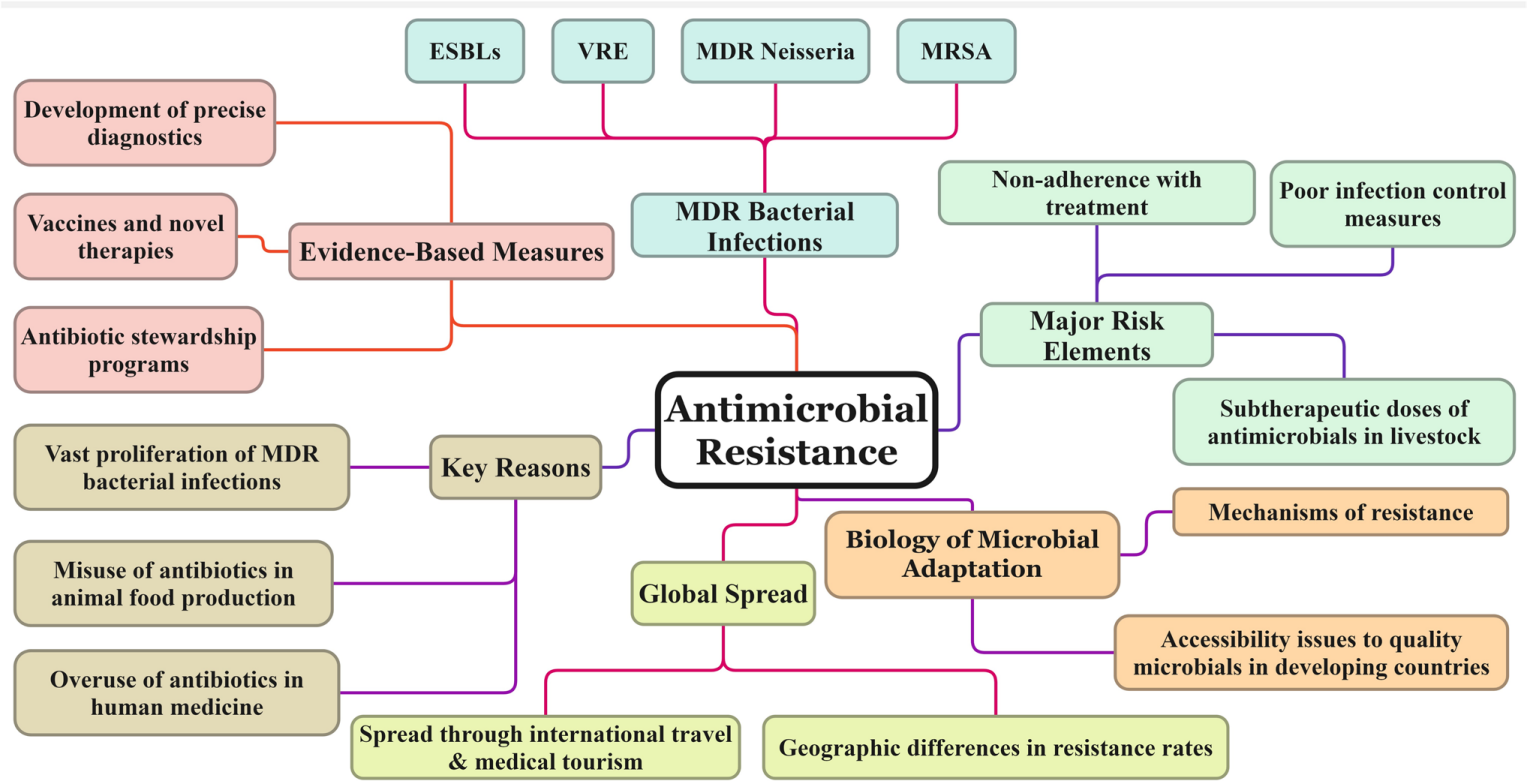
Antimicrobial resistance (AMR) depicts the key reasons, evidence-based measures, and global impact. ESBLs – extended-spectrum beta-lactamases; VRE – vancomycin-resistant enterococci; MDR – multidrug-resistant; MRSA – methicillin-resistant *Staphylococcus aureus.*

As the pace at which antibiotic discovery lags behind resistance, not too many treatment options will remain effective in the coming years, plunging the world into a scenario that looks like a “post-antibiotic era” [27]. Novel economic incentives and policies must be enacted to revitalize depleted antibiotic product development and prioritize using essential drugs for the most critical resistant pathogens [28]. In this light, efficient use of existing drugs could be achieved through disease control, diagnostics tools, and stewardship programs [29]. Dealing with the global menace of antibiotic resistance requires a “One Health” approach across human medicine, agriculture, and the environment. Local and international collective involvement will lead to developing the antibiotics needed to sustain medical treatment of infections in the future, as shown in Fig. 2. Evidence-based measures to prevent and control the spread of MDR pathogens must be combined with antibiotic stewardship programs and the development of more precise diagnostics, vaccines and novel therapies [13, 30, 31].

This review first provides a general overview of bacterial drug resistance and the current challenges in antibiotic drug development, subsequently focusing on novel approaches to antibiotic drug discovery, along with advancements in antibiotic formulation and drug delivery. Furthermore, we briefly discuss examples of recent clinical progress and the translational challenges faced. This review provides a mechanistic insight into the present state of AMR and prospective innovative treatments, thus serving as a comprehensive reference for relevant details and the newest updates on AMR.

## Mechanisms of Antibiotic Resistance

**Fig. 3:**
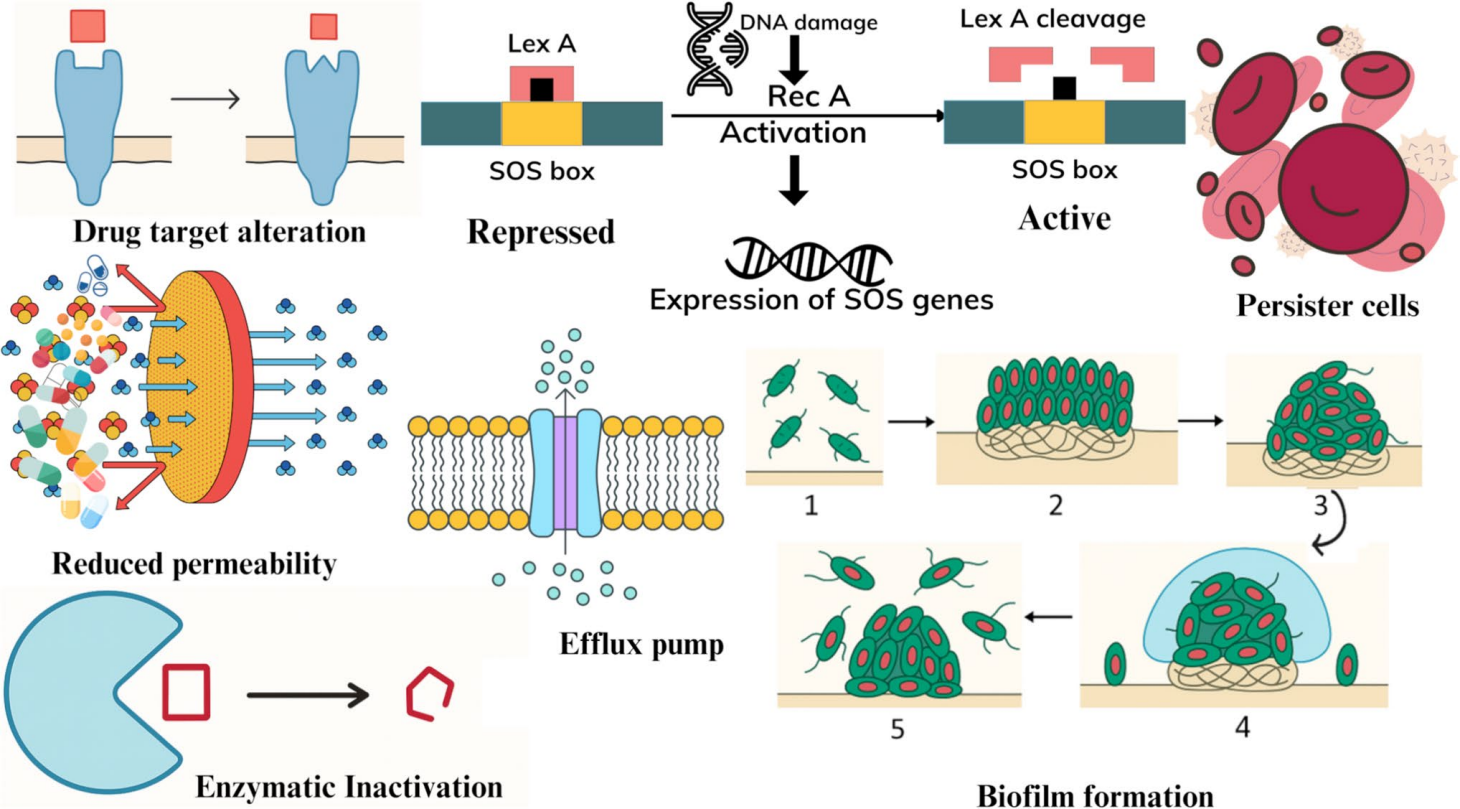
Common mechanisms of antibacterial resistance. RecA – Recombinase A; LexA – LexA Repressor Protein. Stages of biofilm formation: 1. Bacterial cells attach to surface; 2. Irreversible attachment; 3. Bacterial colony formation; 4. Biofilm maturation, 5. Detachment and dispersal of bacterial cells. Images created with Canva

The looming antibiotic resistance that is taking a toll on global health is caused by bacteria avoiding or repelling antibiotics through genetic and phenotypic changes in Table I and Fig. 3. Some bacteria are naturally resistant to certain antibiotics, but others can acquire resistance through mutations or **horizontal gene transfers** via transformation, transduction or conjugation [32, 33]. The resistance genes are spread via plasmids, phages, and efficient mobile elements, enabling bacterial populations to turn into strains that can overcome antibiotic toxicity. For example, MDR plasmids have been widely distributed within Enterobacteriaceae [34].

One crucial resistance mechanism is through **enzymatic inactivation/degradation** wherein bacteria produce enzymes such as β-lactamases and penicillinases to break down antibiotics chemically, inclusive of the penicillins, the cephalosporins, as well as the carbapenems [35, 36]. This results in the drug's ineffectiveness against common pathogens such as Enterobacteriaceae and Pseudomonadaceae*.* Some bacteria can also **change the target sites of antibiotic molecules**, which blocks the natural binding and pharmacological action. For example, there are mutations in penicillin-binding proteins in *Streptococcus pneumoniae* (*S. pneumoniae*) [37] or ribosomal components in *Mycobacterium tuberculosis* (*M. tuberculosis*) [38].

**Table I:** Various Mechanisms by which Bacteria Develop Resistance to Antibiotics

Mechanism	Examples of bacteria	Example antibiotics	Reference
Enzymatic inactivation	Enterobacteriaceae *Staphylococcus aureus*	Penicillins, cephalosporins, carbapenems	[35]
Alteration of drug target	*Streptococcus pneumoniae* *Mycobacterium tuberculosis*	Penicillins, macrolides, aminoglycosides	[37, 38]
Efflux pump	*Escherichia coli* *Pseudomonas aeruginosa*	Fluoroquinolones, tetracyclines, macrolides	[39, 40]
Reduced permeability	*Klebsiella pneumoniae* *M. tuberculosis*	Carbapenems, aminoglycosides, fluoroquinolones	[41, 42]
Biofilm formation	*P. aeruginosa* *Staphylococcus epidermidis*	Aminoglycosides (e.g., tobramycin), β-lactams, and fluoroquinolones	[43, 44]
SOS response	*E. coli* *S. aureus*	Fluoroquinolones	[45]
Formation of persister cells	*M. tuberculosis* *Borrelia burgdorferi*	Rifampicin, isoniazid, pyrazinamide, ethambutol doxycycline, amoxicillin, cefuroxime	[39, 46]

**Efflux pumps** play a prominent role in cell membranes by exporting antibiotics from the cell before reaching their designated targets. This results in reduced efficacy of the antibiotics. AcrAB-TolC in *E. coli* [39] and MexAB-OprM in *P. aeruginosa* [40] are examples of efflux pumps that export antibiotic classes, such as fluoroquinolones and tetracyclines.

Another strategy utilized by the bacteria is **permeability reduction**, where the bacteria adjust porins and cell wall structure to hinder the penetration of antibiotics into the bacterial cell. For example, *K. pneumoniae* porin changes [41] and *M. tuberculosis* cell wall thickening [42] hindered the antibiotic flow into bacteria, resulting in β-lactam resistance. Furthermore, biofilm formation (for example in lungs, catheters, prosthetic joints, and cardiac devices such as pacemakers) helps to clump and shield microbes against numerous antibiotics, preventing even immune attacks from destroying the bacteria. Common bacteria found in biofilms include *Pseudomonas*, which causes pneumonia [43] and *Staphylococcus*, which causes hip joint device infections [44].

The **SOS response** of bacteria is a complex gene network for DNA repair induced by DNA damage [47]. It plays a central role in developing antibiotic resistance, thus increasing the bacterial adaptability [48]. For example, as fluoroquinolone antibiotics are toxic to bacterial DNA, their action can trigger the SOS response among bacteria like *Staphylococcus*, where the bacteria try to fix the broken DNA [45]. However, mutations resulting in antibiotic resistance may happen during the error-prone DNA repair process. One of the key proteins in bacterial DNA repair and SOS response is recombinase A (RecA). RecA facilitates homologous recombination of DNA to repair the damage caused by e.g. antimicrobial agents such as fluoroquinolones [49]. Furthermore, it induces the autocleavage of the SOS repressor LexA, which in turn induces the transcription of SOS genes that regulate e.g. DNA repair and mutagenesis as well as biofilm formation [50].

Moreover, all pathogenic bacteria can form phenotypically non-growing, dormant cells that are tolerant (but not genetically resistant) to antibiotics [39]. Examples of such bacteria that can produce many **persister cells** are *M. tuberculosis* [46] and *Borrelia burgdorferi* (*B. burgdorferi*) [39]. Infections by these pathogens often require extended treatment times [39]. Ultimately, all these mechanisms operate in harmony and transform bacteria via phenotypic and genetic changes to survive antibiotic exposures.

## Challenges in Antibiotics Discovery

### Challenges in the Traditional Antibiotics Discovery Process

**Fig. 4:**
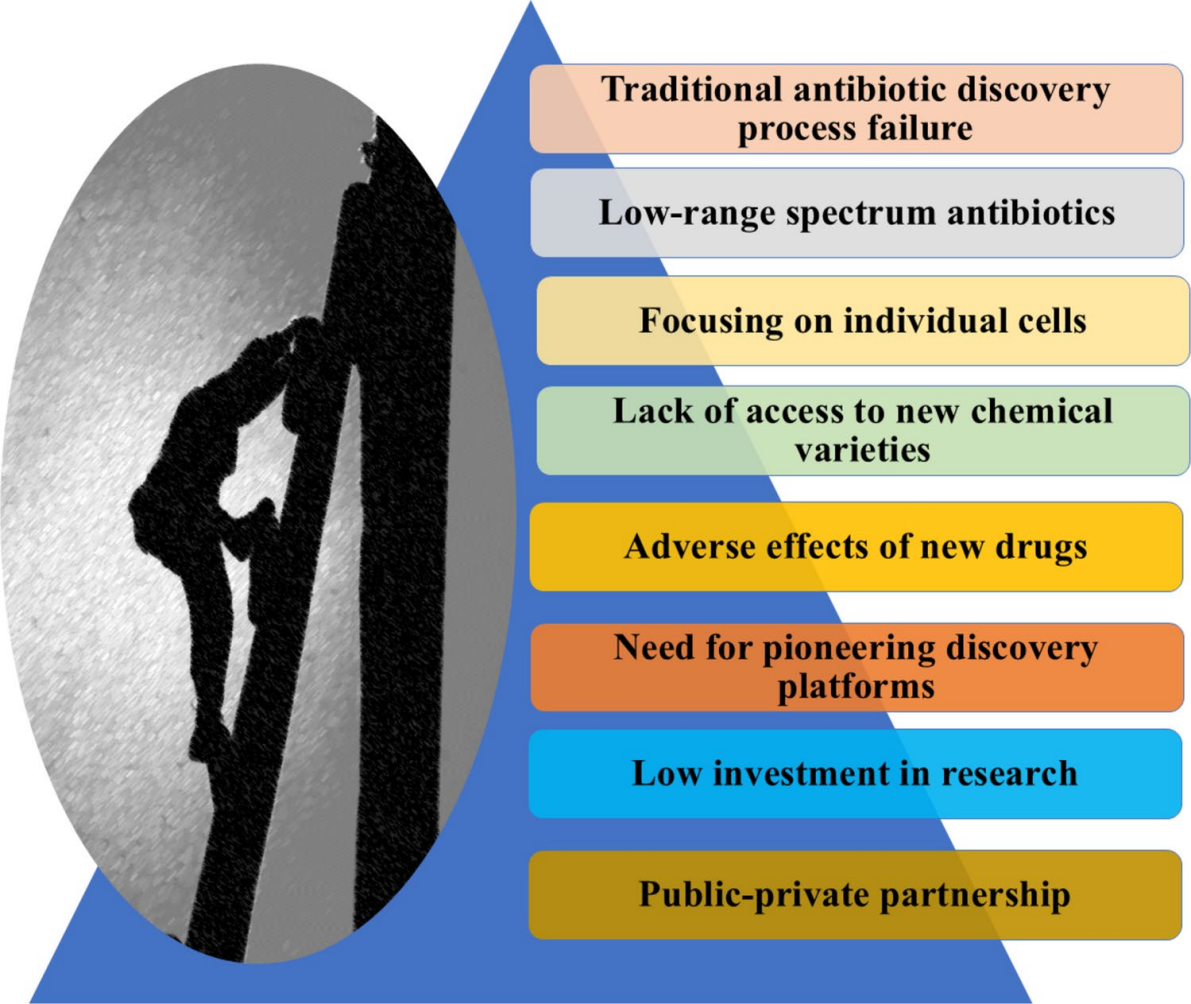
Challenges and limitations in traditional antibiotic discovery

The traditional antibiotic discovery process has several challenges, as shown in Fig. 4. The success rate for discovering and designing effective antibiotic therapeutics has been meager than other therapeutics. The attempts to develop new antibiotics  have been costly and inefficient, as many potential drugs entering clinical trials (90–99%) have been unsuccessful [25, 51]. The antibiotic candidates have failed due to diverse factors such as drug toxicity, inefficient absorption, distribution, metabolism, or excretion (ADME) of the drug, and evolving resistance mechanisms of bacteria [52]. Most antibiotics usually have a low-range spectrum, targeting only some divisions of the bacteria, which helps resistance development as the unaffected pathogens can modify drug targets. At the same time, they remain overall fit [53]. This drives the sectional pressure, eventually benefiting the genetic strains with resistance traits. Furthermore, horizontal gene transfer increases the rate at which the bacteria share antibiotic resistance factors [54]. Accordingly, antibiotics ought to fight an evolutionary arms race against bacteria, which is not a fair battle because bacteria are skillful and can achieve resistance by many methods [55].

There are other problems associated with traditional antibiotic discovery methods, including the search for amplification of known chemical scaffolds, low access to new chemical varieties, and essentially a focus on individual bacterial cells, which may not be effective concerning the bacterial preference of forming colonies and even biofilms with different bacterial species [56]. The more accessible targets may already have been over-exploited. Attempts to overcome resistance entail the creation of drugs that hit new targets, which may lead to more adverse effects and a lack of efficacy in the clinical setting [57]. Traditional antibiotic discovery methods have not been able to eliminate obstacles linked to the pharmacokinetics of antibacterial agents in the last phase of clinical development and the speed of developing antibiotics, which have long-term relevance in clinical practice. This highlights the pressing need to combat anti-microbial resistance through pioneering discovery platforms that have the potential of extending chemical access, finding new bacterial weaknesses, and considering the evolutionary dynamics at the early stages of development. To overcome the present difficulties, it is essential to eliminate diffusing homogeneous and individualistic approaches across business and academia [58].

### Economic Challenges and Lack of Incentives for Pharmaceutical Companies

From an economic point of view, there is very little investment in antibiotic research and development (R&D) from the pharmaceutical industry to allow the development of new antibacterials [59]. One of the main reasons is that antibiotics provide low profit margins. Unlike the long-term use of drugs for chronic diseases, antibiotics are commonly taken only until an infection clears [60]. This phenomenon of lower and shorter-term sales volume makes antibiotics a less profitable investment for pharmaceutical companies. It has become apparent that most antibiotics bring only $100–500 million in peak annual sales, which is insubstantial compared to a new drug for cancer treatment (more than $1 billion in peak annual sales) [26]. Like other drugs, developing new antibiotics is a prolonged, technically challenging, and very expensive process, considering the strict clinical trial requirements. There is, of course, no possibility to make revenue during the development phase. Therefore, the lack of overall profitability and the long R&D timeframes make antibiotics uninteresting to the private sector.

To meet the challenge of this innovation gap, alternative economic models are proposed that will act as the incitement to critical antibiotic research by dividing R&D revenues from sales volumes. For instance, these measures include payment schemes, commercialization awards, patent purchases, and public–private partnerships in which public funding helps R&D at different development stages in pharmaceutical and biotech companies [61]. The funding system and the policy changes of the governments and health institutions will be critical to increasing the production of new antibiotics. Without proper stimuli to pick up the slack in antibiotic R&D, we may again be in the pre-antibiotic era and lose these lifesavers forever.

## Novel Approaches in Antibiotic Discovery

### Exploiting the Genomic Diversity of Bioactive Compound-Producing Bacteria

As we know, bacteria can synthesize and secrete various bioactive compounds, which is worth utilizing in search of novel antibiotics. The genomic potential of Actinobacteria in producing bioactive compounds has recently been investigated and reviewed by Wezel and co-workers [62]. The chemical diversity of the bioactive compounds produced by these bacteria is considerably more than what has been exploited to date [63, 64]. Some specific microenvironment and ecological signals are required to turn on different biosynthetic gene clusters (BGCs), which could be researched by transcriptomics and metabolomics in different environmental assays. Computational tools such as antiSMASH [65], PREDetector [66] or PRISM [67] may be efficiently utilized to detect such BGCs, predict how they are regulated or foresee the structural core of the compound produced by those genes.

Besides, numerous unexplored bacteria are possible sources of various antibiotics. Most species cannot even be cultured under standard laboratory conditions [68]. Teixobactin is an example of an antibiotic compound produced by unculturable soil bacteria, *Eleftheria terrae*, discovered only a decade ago [69]. A technical apparatus called an isolation Chip (iChip) made it possible to cultivate these bacteria and, thus, to isolate and discover a novel class antibiotic, teixobactin [70], which is now in the preclinical stage of development.

### Targeting Novel Bacterial Pathways

Targeting novel bacterial pathways in drug development is another current strategy for combating antibiotic resistance in bacteria. Instead of aiming at inhibition or modulation of the traditional targets of antibiotics, attempts are being made to identify pathways necessary for the microbes’ virulence (i.e., ability to cause a disease or damage) and the pathogenesis (how the infection develops to a disease) [71]. One such approach is to interfere with quorum sensing (QS), the bacterial communication method used for coordinated processes, such as biofilm formation or enhancement of virulence [72].

Several QS inhibitors (QSIs) or quenchers (QQs) have been discovered/developed, for example, against *P. aeruginosa* [73] and *S. aureus* [56]. Another strategy is to destroy the primary defense, i.e., bacteria's cell wall, through novel targets, different from the target of the commonly used β-lactam antibiotics [74, 75]. Moreover, exploiting the peptidoglycan degrading enzymes of bacteriophages provides a promising approach to kill Gram-positive bacteria [76, 77].

It should also be mentioned that the prokaryotic cell membrane can be considered as an additional target for antibiotics [78]. Besides antimicrobial peptides, synthetic amphiphilic polymer constituents can also be designed to disrupt bacterial membranes, leading to the efflux of intracellular substances and thus affecting the bacteria’s ability to survive [79]. The second line of defense in bacteria is the active expulsion of strong antibiotics through the cell wall via the cell membrane. Efflux pump inhibitors are being developed as an adjunct therapy to prevent these pumps from doing their job and bring back the susceptibility to antibiotics [80].

Furthermore, essential metabolic pathways of bacteria, such as folate biosynthesis and amino acid metabolism, provide potential novel targets for antibiotic development. Trimethoprim-sulfonamide combinations have already been used for decades to outcompete bacterial folate synthesis. Initially, it was hoped that the combination would protect both drugs against the development of resistance, but unfortunately, that has not been the case [81, 82], so novel approaches are needed. On the other hand, preventing the specific biosynthesis of amino acids such as lysine may be a proper antibacterial strategy [83, 84].

In addition, bacteria's stress response mechanisms can be inhibited to make them more vulnerable to antibiotics-induced damage. Here, we refer to small molecules used as inhibitors of bacterial heat shock proteins or oxidative stress responses of this type [85]. Moreover, a direct targeting approach may stimulate the rapid release of bacterial endotoxins. Such toxin release also occurs at the last phase of an infection, where the immune mechanisms clear the pathogens [86]. In summary, the variety of non-traditional approaches to target resistant bacteria in Table II gives us hope for fighting antibiotic resistance globally. In the next paragraph, the antivirulence approach is given a more detailed look at current examples of antivirulence compounds in the clinic that are under development.

**Table II:** Strategies for Combating Multidrug-resistant Bacteria

Approach	Description	Example applications	Reference
Inhibition of quorum sensing	Target bacterial communication systems involved in quorum sensing to disrupt cell-to-cell signaling, biofilm formation, and virulence factor production	Small molecule inhibitors of quorum sensing in *P. aeruginosa* Natural compounds targeting quorum sensing in *S. aureus*	[56, 73]
Interference with bacterial cell wall	Develop compounds that disrupt bacterial cell wall synthesis or integrity, leading to cell lysis and death	Inhibitors of peptidoglycan biosynthesis Lytic enzymes targeting cell wall components	[76, 77]
Disruption of bacterial membrane function	Design molecules that disrupt bacterial membrane integrity or function, causing leakage of intracellular contents and cell death	Antimicrobial peptides targeting bacterial membranes Amphiphilic polymers are disrupting membrane integrity	[79]
Inhibition of bacterial efflux pumps	Block bacterial efflux pumps expel antibiotics from the bacterial cell and restore antibiotic susceptibility	Efflux pump inhibitors as adjuvants to existing antibiotics Small molecules targeting specific efflux pump proteins	[80]
Interference with bacterial metabolism	Target essential metabolic pathways in bacteria, such as folate biosynthesis or amino acid metabolism, to disrupt bacterial growth and survival	Trimethoprim-sulfamethoxazole combination targeting folate biosynthesis Inhibitors of bacterial amino acid biosynthesis	[81, 82]
Modulation of bacterial stress responses	Develop compounds that interfere with bacterial stress response pathways, making bacteria more susceptible to antibiotic-induced stress and cell death	Small molecules targeting bacterial heat shock proteins Compounds inhibiting bacterial oxidative stress response pathways	[85]
Blocking bacterial virulence factors or their production	Inhibit the synthesis or activity of bacterial virulence factors involved in pathogenesis, attenuating bacterial virulence and enhancing host immune responses	Inhibitors of bacterial toxins or adhesins Small molecules targeting bacterial secretion systems	[87, 88]

### Antivirulence Approach

As the name of the approach reveals, the antivirulence strategy aims to prevent the bacterium’s pathogenicity (virulence) rather than the bacterium’s viability. Therefore, in contrast to most conventional antibiotics that perturb the homeostasis of normal microbiota, the antivirulence approach intends to target and inhibit the molecules and structures vital for bacteria to inflict disease [89]. This specificity contributes to the fact that only pathogenic bacteria are eliminated, and the beneficial bacteria remain almost untouched, reducing the risks of adverse effects. The suppression of virulence could enable the host’s immune response to prevent bacteria from colonizing the body or more efficiently clear any established infections [90]. This strategy is assumed to act to reduce the selective pressure for resistance development since exposure has no consequence on bacterial survival. Therefore, the approach can help extend the shelf life of existing antibiotics and develop a longer-term approach to performing antibacterial therapy [91].

Recent research has discovered several antivirulence agents; some are already in clinical use, and numerous are under development for representative examples in Table III. These antivirulence molecules target various bacterial mechanisms, including direct neutralization of virulence factors, inhibition of their secretion, interfering with bacterial signaling, preventing biofilm formation, reversal of resistance, and suppressing virulence factor expression [87, 88]. For example, raxibacumab and obiltoxaximab are approved monoclonal antibodies targeting the protective antigen of *Bacillus anthracis* (*B. anthracis*) and are utilized for treating anthrax infections. Suvratoxumab and shigamab are among those that are still under development, and both target toxins. Suvratoxumab is a monoclonal antibody targeting *S*. *aureus* α‐hemolysin (Hla), which has shown some efficacy in treating ventilator-associated pneumonia [92]. Shigamab comprises two antibodies against shigatoxin 1 and 2 (Stx1 and Stx2), which Shiga-toxin-secreting *E. coli* produces.

Some examples targeting the type III secretion system (T3SS) in different gram-negative bacteria include fluorothiazinone, aurodox, and gremubamab [93]. T3SS is an elegant molecular syringe for protein secretion in several gram-negative bacteria. It helps the bacterium to inject different effector proteins into target host cells. These proteins can regulate the functions of host cells and support the bacteria in their effect of immune escape and colonization. T3SS significantly contributes to the virulence of various pathogens, including *Salmonella, Shigella, Yersinia* and *E. coli*. It is a broad-spectrum target against several virulence factors. The action of gremubamab is bivalent in that it targets not only the TS33 but also the adhesin Psl exopolysaccharide in *P*. *aeruginosa*.

LED209 is also a virulence inhibitor of gram-negative bacteria [89]. It targets the histidine sensor kinase QseC, a receptor in a broad spectrum of pathogens, inhibiting downstream QseC-mediated production of virulence factors. Although LED209 significantly reduced bacterial virulence *in vitro*, it failed to demonstrate efficacy in animal models because of its rapid entry into the circulatory system [94]. Nevertheless, LED209 serves as a proof of concept for targeting QseC, allowing inhibition of virulence without directly affecting bacterial growth. The compound has been studied further [95], and it was recently shown that when combined with cellulose membrane, it can prevent enterohemorrhagic *E. coli* (EHEC) adhesion and reduce biofilm formation [96, 97].

M64 and MAC-545496 are two recently identified compounds currently in preclinical development, and both target bacterial biofilm formation. M64 binds to the quorum-sensing regulator MvfR, disrupting biofilm formation in *P. aeruginosa* and enhancing the antibiofilm activity of antibiotics [98]. MAC-545496 inhibits the *S. aureus* biofilm formation and reverses β-lactam resistance in MRSA by inhibiting the glycopeptide-resistance-associated protein R (GraR) [99]. A very recently reported synthetic, antibacterial molecule MTEBT-3 has also been shown to inhibit *P. aeruginosa* and carbapenem-resistant *K. pneumoniae* biofilm formation, which is likely related to its ability to suppress the expression of certain key virulence factors [33, 100]. These drugs exemplify how targeting specific virulence factors can disarm pathogens while boosting the efficacy of existing antibiotics. For instance, fluorothiazinone, in combination with cefepime, has shown promising results in treating complicated urinary tract infections [101].

**Table III:** Examples of Antivirulence Drugs Approved into the Clinic or Currently under Development

Compound	Type	Target	Target pathogen	Status	Reference
Raxibacumab	mAb IgG1	PA	*Bacillus anthracis*	FDA-approved (2012)	[102]
**Obiltoxaximab (ETI-204)**	mAb IgG1	PA	*B. anthracis*	FDA-approved (2016)	[103]
Gremubamab (MEDI3902)	mAb	Psl & PcrV	*P. aeruginosa*	Phase II	[93]
**Shigamab (cαStx1/cαStx2)**	mAb	Stx1 & Stx2	*E. coli*	Phase II	[104]
Suvratoxumab (MEDI4893)	mAb	Hla	*S. aureus*	Phase II	[105]
**Fluorothiazinone**	Small molecule	T3SS	Gram-negative bacteria	Phase II	[63]
Aurodox -derivates	Natural product	T3SS-related genes	Enteropathogenic & enterohemorrhagic *E. coli*	Preclinical	[92, 106]
**LED209**	Small molecule	QseC	Gram-negative bacteria	Preclinical	[89]
M64	Small molecule	MvfR	*P. aeruginosa*	Preclinical	[107]
**MAC-545496**	Small molecule	GraR	*S. aureus*	Preclinical	[99]

PA, Protective antigen; PsI, exopolysaccharide Psl; PcrV, T3SS type PcrV; Stx2, shigatoxin type 2; Hla, α‐hemolysin toxin; T3SS, type III secretion system; QseC, histidine kinase; MvfR, Multiple Virulence Factor Regulator*;* GraR; glycopeptide-resistance-associated protein R

### Harnessing the Microbiome

The microbiome of human beings has a crucial role in protecting, dominating pathogens, controlling immune function and contributing to the secretion of antimicrobial compounds. Advances  in DNA sequencing have revealed microbial genetic diversity and these microbiomes' ability to prime human health [108, 109]. This vast amount of genetic data and microbial capabilities could thus empower the manufacturing of microbiomes being used more precisely to avoid, rather than recover from infections, without antibiotics [110].

Applying probiotics constitutes methods like rendering pathogenic organisms out of competition and strengthening the human body's immune system [111]. *Clostridioides difficile* (*C. difficile*) infection damages the gut microbiome in patients. A healthy individual's fecal microbiota transplantation (FMT) has proven effective in balancing and restoring the gut microbiome. The aim is to restore the healthy microbiome to outcompete and replace the *C. difficile* bacteria causing colon infection [112, 113].

In addition, increasing abilities by creating a new field of synthetic biology opens additional opportunities in designing microorganisms with inherent therapeutic properties [114]. Such living medicines have been engineered by scientists to blackout information concerning pathogen presence, transport antimicrobial agents to required locations and inhibit molecular conduits in pathogens. Such probiotics have synthetic gene circuitries that act as an interface for controlling activity with the help of “gene chisellers” [115]. Thus, by studying the microorganisms with antibiotic properties produced by the biome, more microspecies will be identified for the administration of probiotic therapy [116, 117].

An encouraging example of successful mining of the microbiota for antibacterial properties was the identification of lugdunin, a peptide antibiotic. It was first described in 2016 when a team of researchers from the University of Tübingen in Germany wanted to study the microbiota in noses [118]. Specifically, they isolated nasal swabs and grew skin bacteria *Staphylococcus lugdunensis*, often found in human specimens. The contents of the culture broth were then characterized by mass spectrometry [119]. This analysis provided an unknown compound that was then purified and identified. The German researchers named this new thiazolidine-containing cyclic peptide “lugdunin”, the name derived from the Latin name of their town. Using lugdunin, bacterial growth could be effectively stopped *in vitro*, and lugdunin appears to be effective against human pathogens such as *S. aureus* and *Enterococcus faecalis* (*E. faecalis*) [120]. It was found that lugdunin can eradicate *S. aureus* and the antibiotic-resistant strain by demolishing their bacterial cell membranes.

Moreover, it was shown that lugdunin disrupted the cellular compartment in *E. faecalis,* leading to cell lysis and death. Subsequent studies have revealed that the dose of lugdunin needed to prevent bacterial growth is comparable to that of the common antibiotic drugs. In the mice experiment with MRSA infection, lugdunin diminished the bacterial count to the same level as vancomycin, a broad-spectrum antibiotic for MRSA [121]. Further, the bacterial cells did not develop any resistance to lugdunin throughout the long-time treatment. The above positive findings indicate that lugdunin may one day help treat antibiotic-resistant bacterial infections.

Finally, all antibiotic resistance may originate from the microbiome, suggesting that solutions might be found by embracing this field. Limitations of modern technology hinder the immediate integration of advancements in microbiome science expected in the future. However, as scientific understanding of the microbiome progresses, this knowledge could facilitate a shift from broad antibiotic use to targeted ecological approaches that promote microbial symbiosis.

### Repurposing Existing Compounds

Drug repurposing or repositioning is the most powerful currently known approach for finding new drugs for various indications. It is a cheaper and less time-consuming process than developing a new drug. The FDA has also accepted this approach. Repositioned drugs thus have the built-in advantages of safety and clearly defined dosing, which may expedite a drug’s regulatory process [122].

**Fig. 5:**
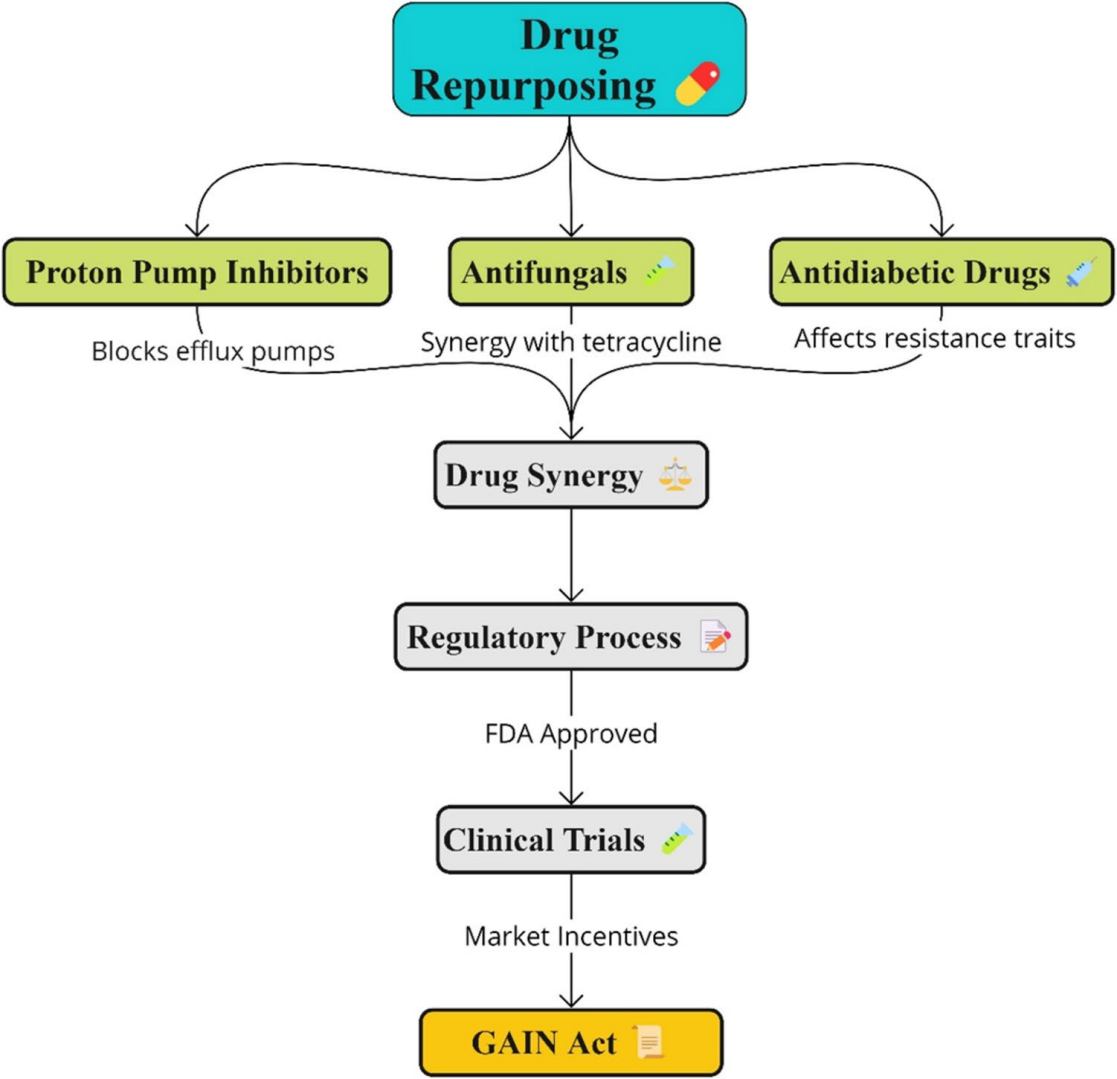
Repurposing of existing compounds

Some seemingly non-antibiotic substances have shown positive outcomes as they have bactericidal properties or increase the susceptibility of MDR bacteria toward antibiotics and the host’s immune response in Fig. 5. These include proton pump inhibitors (PPIs), antifungals, anti-inflammatory agents, antidiabetics, and antihistamines [121]. For instance, omeprazole, a PPI, has been identified to block efflux pumps in gram-negative bacteria, making the bacteria sensitive again to antibiotics such as ciprofloxacin. It is postulated that some drugs target the evolutionary traits of antibiotic-resistant strains. For example, the antidiabetic drug metformin affects traits or adaptations that make bacterial strains resistant to aminoglycosides [123].

Drug combinations are especially effective in defeating resistance when the repurposed drugs synergize with antibiotic substances. For example, the antifungal fluconazole synergized in antibacterial activity against carbapenem-resistant *K. pneumoniae* with tetracycline [124]. Also, combining the antihistamine chlorcyclizine and the topical antiseptic chlorhexidine has *in vitro* antibacterial activity with trimethoprim and ampicillin [12, 125].

Many antiviral agents have shown a promising range of antibacterial effects in terms of efficacy and safety benefits based on retrospective analyses and retrospective repurposing of compounds screened from various libraries. It is still necessary to carry out prospective interventional clinical trials to be able to make decisions based on specific guidelines. The adverse effects/risks and the pharmacokinetics/systemic exposure must be considered, especially if the drugs are combined [126]. Thus, clinical trials can be conducted using topical or oral routes of administration to avoid high systemic exposure to the drug or compound under investigation [127].

Regulatory concerns regarding the repurposing of drugs for combating antibacterial resistance are needed. The costs arising from the regulatory challenges involved in drug repurposing and the unpredictability of the future financial profitability of new clinical trials may deter drug developers from further researching existing drugs [128]. The promise of more extended market exclusivity periods and patent protections through policy changes like the US Generating Antibiotic Incentives Now (GAIN) Act may encourage developers to bear the risks and costs of new clinical trials necessary to revisit existing drugs [129]. The GAIN Act seeks to streamline the approval process for these repurposed drugs so that they can be given market clearance.

## Advancements in Antibiotic Formulation for Improved Delivery

Several strategies are currently employed to deliver and formulate antibiotics, potentially improving antibacterial efficacy and sensitivity toward resistance. Some representative examples have been outlined in Table IV.

**Table IV:** Highlights of Innovative Approaches to Antibiotic Delivery and Formulation

Approach	Description	Examples	Reference
Nanoparticle-based delivery systems	Utilizing nanoparticles as carriers for antibiotic drugs, improving drug stability, increasing bioavailability, and enabling targeted delivery	Lipid nanoparticles Polymeric nanoparticles, e.g. poly(lactic-co-glycolic) acid, PLGA	[130, 131]
Inherently antibacterial nanoparticles	Unique particles with inherent antimicrobial ability can eliminate bacteria or prevent their reproduction without applying chemical agents. Since they reduce infection, they are used in diverse products, from medical devices to wound dressings	Silver nanoparticles (AgNPs) Metal and metal oxide nanoparticles, e.g., ZnO NPs Carbon dots, carbon quantum dots (CQDs), metal–organic frameworks (MOFs)	[132–135]
Liposomal formulations	Encapsulating antibiotics within lipid bilayers enhances drug solubility, stability, and tissue penetration while reducing toxicity	Liposomal amikacin for treating pulmonary infections Liposomal vancomycin for treating Gram-positive bacterial infections	[136]
Polymeric micelles	Forming self-assembled structures composed of amphiphilic polymers to encapsulate hydrophobic antibiotic drugs, improving solubility and delivery	Polymeric micelles loaded with ciprofloxacin for enhanced bioavailability (Pluronic F127-based micelles)	[137]
Nanosuspensions	Producing submicron-sized drug particles dispersed in an aqueous medium, increasing drug surface area and dissolution rate for improved absorption	Cefuroxime axetil nanosuspension for oral delivery Itraconazole nanosuspension for intravenous administration	[138, 139]
Microparticle depots	Developing biodegradable microparticles or implants for sustained release of antibiotics, providing prolonged therapeutic effect and reducing dosing frequency	Biodegradable polymer (polycaprolactone) microparticles loaded with gentamicin for local delivery in bone infections	[140–143]
Combination therapies	Combining antibiotics with adjuvants or synergistic compounds enhances antibacterial activity, overcomes resistance mechanisms, and reduces side effects	Antibiotic-probiotic combinations for treating gastrointestinal infections Antibiotic-metal NP combinations for enhanced bactericidal activity (silver nanoparticles and antibiotics)	[91, 144]
Inhalable antibiotics	Formulating antibiotics as dry powders or aerosols for inhalation, targeting respiratory infections and improving drug delivery to the lungs	Tobramycin dry powder inhalation for treating cystic fibrosis-associated lung infections Colistin inhalation therapy for pneumonia	[145, 146]
DNA nanotechnology	DNA sequence modified by aptamer could specifically recognize and bind to the target RNA, restoring the sensitivity of antibiotics to multi-resistant bacteria	DNA nanoflower nanoparticles	[147]

### Nanotechnology-Based Approaches to Antibiotic Drug Delivery

**Fig. 6:**
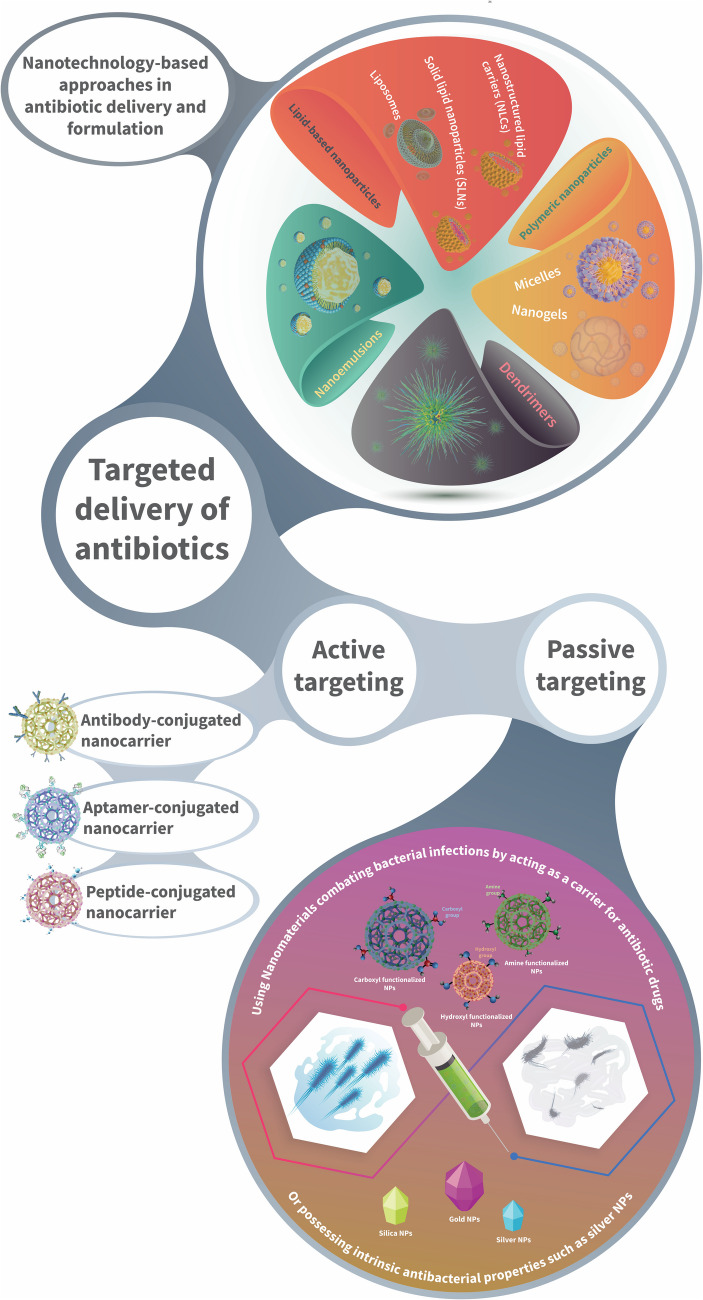
Illustration of the advancements in antibiotic delivery and formulation, classified into nanotechnology-based and targeted antibiotic delivery

In recent years, the preparation and application of new methods for the delivery of antibiotics have risen considerably, particularly in the incorporation of nanotechnology in molecular recognition processes since nanoparticles (NPs) hold specific physicochemical properties that can be tailored to fit specific applications [148]. As in other therapeutic areas, NPs as drug carriers has shown to be able to enhance the performance and efficiency also of conventional antibiotics. As a result, NPs can improve the stability of antibiotic drugs and raise their concentration at the target site, targeting performance and transport drugs primarily to the site of infection. They can also prolong the release of antibiotics, providing a platform for sustained treatment for a more extended period [27]. An illustration of different nanotechnologies employed for antibiotic delivery are presented in Fig. 6 and outlined in the below section.

#### Lipid-based nanoparticles

Encapsulation appears as a persuading method of preserving antibiotics from severe processing and storage conditions and providing efficient formulations. Recently, nanocarriers such as liposomes, solid lipid nanoparticles (SLNs), and nanostructured lipid carriers (NLCs) have been efficiently proven successful for antibiotic delivery and controlled release [130, 149, 150].

Nanoparticles of these materials have been employed to deliver antimicrobial peptides, bacteriocins, enzymes, essential oils, and antimicrobial agents of plant origin. Most of the investigations continued with liposomes while exploring other physiochemical characteristics and applications of SLNs and NLCs in the delivery of antimicrobial substances. Research indicates lipid-based formulations can enhance the characteristics of antimicrobials and improve effectiveness in fighting microbial pathogens [151].

Werner *et al.* (2024) focused on overcoming the challenges associated with the oral delivery of the vancomycin derivative FU002, which has poor bioavailability. The study developed a liposomal nanocarrier formulation to enhance the oral delivery of FU002. The liposomes were modified with cell-penetrating peptides on their surface to improve their binding and permeation capabilities. The results demonstrated that the liposomal formulation effectively binds to CaCO‐2 cells without causing cytotoxic effects. *In vivo*, studies revealed that liposomal FU002 increased oral bioavailability compared to free drugs. The liposomal formulation of FU002 maintains its antibacterial activity both *in vitro* and *in vivo*. When given orally, liposomal FU002 significantly treats a murine systemic infection model. The unbound and CPP-GCTE-encapsulated FU002 MIC test was conducted to assess MRSA and vancomycin-resistant *Enterococcus faecium*. The results showed that CPP-GCTE-liposomal FU002 at 1µg/mL and 0.75 µg/mL had a lower MIC compared to free FU002 at 2 and 1 µg/mL against *S. aureus* and *Enterococcus faecium* [152].

In 2019, Ghaderkhani and colleagues evaluated rifampicin-loaded solid lipid nanoparticles (Rif-SLNs) against *Brucella abortus*. The prepared SLNs had an average diameter of 320 nm with a spherical shape. Both free Rif’s solution and Rif-SLNs exhibited MIC values of 8 µg/mL and 4 µg/mL, respectively, suggesting the superior effectiveness of Rif-SLNs compared to free rifampicin. Phagocytic cells can naturally ingest SLNs, which helps deliver drugs to specific targets and improves their efficacy by releasing their contents directly into the target cells. Moreover, SLNs address limitations of Rif, such as solubility and absorption, by enhancing its stability and permeability in intracellular environments [153].

Alavi *et al.* (2022) attempted to improve the oral efficacy of antibiotics, the most popular method for treating MRSA skin infections, because of their high patient compliance and preference. They fabricated a PEGylated-NLC known as PEG-TMP/SMZ-NLC, with a particle size of 187 ± 9 nm and a high drug encapsulation efficiency (93.3%). Experimental results showed a 2.4-fold decrease in the drug’s toxicity and an eightfold increase in antibacterial properties with PEG-TMP/SMZ-NLC. *In vivo* studies on mice infected with MRSA suggested a fivefold reduction in drug side effects and a 10 000-fold enhancement in antibacterial effect. The obtained results demonstrate the capability of PEGylated NLCs to improve the oral bioavailability of antibiotics and their effectiveness in treating MRSA skin infections [154].

Selvadoss with team developed liposomal oleic acid (LipoOA) nanoparticles loaded with antibiotics to treat multidrug-resistant *P. aeruginosa*. The findings indicated that resistant strains had MIC values above 1024 μg/mL for free ampicillin (AMP) and cephalexin (CN). Nevertheless, LipoOA loaded with AMP and CN significantly reduced the MIC levels to 8–16 μg/mL. Similarly, the MIC of free ceftazidime (CAZ) varied between 32–512 μg/mL, whereas LipoOA-CAZ decreased MIC to 2–8 μg/mL [155].

#### Polymeric nanoparticles

Polymeric nanoparticles, including polymeric micelles and nanogels, can be prepared with different physicochemical properties, such as size, shape, surface charge, and composition. In 2023, Zlotnikov *et al.* [156] investigated polymeric micelles as potential drug carriers for the targeted delivery of moxifloxacin. Chitosan and cyclodextrin modified with oleic acid, were used as the hydrophilic components of the micelles. The micelles were prepared with varying degrees of modification to achieve an optimal grafting degree of 15–30% regarding the lowest critical micelle concentration (CMC). They exhibited a hydrodynamic diameter of 60–100 nm. The results from the *in vivo* study indicate that the antibiotic's activity remains effective for over four days when delivered in micellar systems.

In contrast, the activity of the free-form antibiotic decreases after two days. Pharmacokinetic experiments revealed that moxifloxacin delivered in micellar systems exhibited 1.7 times greater efficiency than the free-form antibiotic. The study demonstrated enhanced antibacterial effects, increased penetration into bacterial cells, and prolonged drug activity compared to its free form, making it a potential treatment for combating resistant bacterial infections and multidrug resistance [156].

In 2021, Antonia Tănase *et al.* developed mixed Pluronic (F-127)-Cremophor polymeric micelles for poorly soluble antibiotic norfloxacin. The optimal surfactant ratio was determined to reduce the CMC. The mixed micelles showed extended drug release and good biocompatibility with fibroblast cells. The outcomes showed that the norfloxacin-loaded mixed micelles successfully killed both *E. coli* (ATCC 25922) and clinical strains, displaying MIC values of 0.039 µg/mL and 0.078 µg/mL, respectively [157].

In 2020, Guo *et al.* explored the effectiveness of hypocretin A (HA) loaded lipase-sensitive polymer micelles for treating MRSA infections. A nanocarrier system was created by utilizing mPEG-PCL micelles loaded with HA, for which the release is driven by the hydrolysis of ester bonds in PCL by lipase. Using photodynamic therapy, the micelles showed strong anti-MRSA effects in both laboratory and living organism settings. Upon contact with lipase-secreting bacteria, the micelles released HA due to the degradation of the PCL core. The HA-loaded micelles demonstrated low hemolytic activity, good biocompatibility, and improved water solubility of HA. In a model of acute peritonitis, the micelles notably boosted the survival rate of mice that received treatment. The results showed that under exposure to light, mPEG-PCL/HA micelles had a MIC of 0.69 mg/L (HA concentration) and a minimum bactericidal concentration (MBC) of 1.38 mg/L (HA concentration). This research emphasizes the possibility of utilizing lipase-responsive polymer micelles as a nanocarrier platform for successfully managing MRSA infections [158].

Subsequently, Guangyue Zu and colleagues created a nanogel with antibacterial properties to deliver triclosan. The nanogel design includes poly(N-isopropylacrylamide-co–N-[3-(dimethylamino)-propyl]methacrylamide) and is altered with 1-bromododecane to impart hydrophobicity to enhance triclosan loading. The nanogel interacts efficiently with the bacterial cell wall, causing the membrane to break down and allowing triclosan to enter the bacterial cell effectively. The antibacterial impacts of various nanogels were evaluated on Gram-positive bacterial strains. All the blank nanogels had no effect except for 1-bromododecane (Qc12-NG) nanogel, which showed antibacterial activity against *Staphylococcus epidermidis* (HBH 45) and other strains at MIC values of 500 μg/mL. The bactericidal effects of free tricosan against all bacterial strains were reported, with MIC values of 1.25 μg/mL and 2.5 μg/mL. Triclosan-infused nanogel (Qc12-NG + T) demonstrated higher antibacterial effectiveness, with MIC values of 2.46 ng/mL for *S. epidermidis* (ATCC 12228) and *S. aureus* (5298) and 4.91 ng/mL for *S. epidermidis* (HBH 45) and *S. aureus* (ATCC 12600) [159].

#### Dendrimers

Dendrimers, considered nanostructures, have also sparked attention in antibiotic delivery. These are large molecules with much branching, and each manufacturer has an accurate molecular form, and the size and surface chemistry can be controlled during its production. This feature enables them to effectively control the respective constitution and surface properties, resulting in improved drug loading, targeting capabilities and higher solubility of the drugs [160]. Dendrimers can overcome antibiotic resistance mechanisms by disrupting biofilms and inhibiting efflux pumps.

Moreover, certain types of dendrimers are compatible with living organisms and biodegradable, making them appropriate for use in medical areas [161]. Liu *et al.* developed platensimycin (PTM)-encapsulated poly(lactic-co-glycolic acid) (PLGA) and poly(amidoamine) (PAMAM) dendrimeric nanoparticles for treatment against MRSA biofilm formation. PLGA/PTM and PAMAM/PTM NPs exhibit an MIC range of 0.125 − 1 μg/mL against MRSA after 12 or 24 h. Furthermore, the inhibiting of biofilm formation was successful with PLGA/PTM and PAMAM/PTM nanoparticles. PAMAM/PTM NPs and PLGA/PTM NPs at 0.125 μg/mL and 0.25 μg/mL decreased 95.0% and 93.9% in biofilm formation, respectively. Also, PTM, at 0.5 μg/mL, inhibited biofilm growth by 88.3%. 128 μg/mL PAMAM dendrimer resulted in a 30% decrease in biofilm formation [162].

In 2019, Maji *et al.* developed pH-responsive lipid-dendrimer hybrid nanoparticles (LDH-NPs) for targeted delivery of vancomycin (VCM) to intracellular bacterial pathogens. The LDH-NP surface charge was altered in acidic pH from negative to positive. Drug release experiments demonstrated faster VCM release at pH 6.0 compared to pH 7.4. VCM-loaded LDH-NPs exhibited eightfold lower MICs against MRSA at pH 6.0 and 7.4 than free VCM. Cellular studies confirmed significant intracellular accumulation of LDH-NPs, leading to adequate clearance of intracellular bacteria [163]*.*

#### Nanoemulsions

Nanoemulsions are widely recognized as a potential type of transport for antimicrobial medications. They have many beneficial traits that make them appealing, such as high stability for long-term storage, easy synthesis for efficient production, and better drug absorption than traditional formulations [164, 165]. Nanoemulsion improves stability and bioavailability by increasing the solubility of poorly water-soluble antibiotics and enhancing the surface area for interaction with bacteria, facilitating deeper penetration into bacterial biofilms and tissues. This enhanced penetration increases local antibiotic concentrations at infection sites and improves treatment efficacy [166, 167].

However, the magnitude of surface charge can also significantly affect the stability of nanoemulsions. The use of nanoparticles with negative charges is enhanced in terms of stability in the solution and minimum aggregative potentials to afford the necessary concentration of the antibiotics for as long as is required. [166]. Alarjani *et al.* investigated the antimicrobial function of propolis concentrations loaded onto chitosan nanoemulsion that exhibited significant antibacterial activity against MRSA at 150 ng concentration with an inhibition zone of 31.05 mm. In contrast, the 150 ng free antibiotic inhibition zone was 11.21 mm [168].

Haddaji *et al.* evaluated the antimicrobial properties of biosurfactant nanoemulsion (BNE). According to the results, the MIC and MBC of *Bacillus* sp. crude biosurfactants and their nanoemulsions were used to assess their effectiveness against *S. aureus* and *E. coli* pathogenic bacteria. The *Bacillus* sp. crude biosurfactant showed MIC values between 4 and 2 mg/mL, with MBC values around double the MIC values. Experiments were also conducted to evaluate the anti-biofilm formation ability of nanoemulsions by assessing their ability to prevent biofilm attachment on the surface. The results demonstrated significant antibiofilm activity of the biosurfactant against tested bacterial strains. This effect was notably enhanced when crude biosurfactant nanoemulsions were used [169].

#### DNA nanotechnology

DNA (deoxyribonucleic acid) is a molecule encoding genetic information biologically. DNA sequences strictly follow the Watson–Crick base pairing principle. DNA sequences are self-assembling, highly programmable and predictable, making them suitable for constructing DNA nanostructures [170]. Organizing definite sequences to build various two-dimensional and three-dimensional formations, such as origami and tetrahedra, is possible. DNA nanotechnology is highly versatile in its capabilities; it can be used to improve drug targeting, drug delivery and constructing intelligent materials, and it is essential in many biosensors [171].

Furthermore, natural DNA materials are biocompatible and biodegradable, which is essential considering toxicological and sustainability issues. For example, aptamer-based DNA nanotechnology shows excellent potential in identifying bacteria (diagnostics), antibacterial treatment and biofilm eradication, thus presenting novel approaches to combat MDR bacteria. Aptamers are small nucleic acid molecules that selectively bind to target molecules. They are equipped with particular three-dimensional conformations and seem to demonstrate better target affinity and even lower concerns of immunogenicity compared to antibody therapeutics [172]. Aptamers are thermostable and chemically stable, and chemical modification can enable their conjugation to nanoparticles, drugs, or other nucleic acid drugs to improve cellular uptake. They can effectively interact with the cell surface receptor molecules, thus selectively delivering therapeutic agents to the target cells [173].

Also, DNAzymes (deoxyribozymes) coupled with nanotechnology can be used to construct efficient drug delivery systems [174]. DNAzyme is a single-stranded deoxynucleotide with catalytic activity for cleaving the target RNA after the hybridization of the RNA to the deoxynucleotide [175]. Its functional parts include the recognition arm to selectively bind the target RNA, the catalytic core to employ magnesium ions, and other assisting factors to activate water molecules, which attack and cleave the target phosphodiester bond of the RNA. DNAzymes are appreciated mainly because of their high stability and because they are easily programmable and cost-efficient. Regarding drug-resistant bacteria, DNAzymes can reactivate bacteria’s susceptibility to antibiotics by modulating gene expression and synthesis/polysynthesis of drug-resistant bacteria to prevent their reproduction.

For instance, Ran *et al.* applied isothermal rolling circle amplification (RCA) technology to design a DNA nanoflower carrier to target bacterial keratitis caused by MRSA [176]. With the aid of Mg2 +, the DNAzyme released from the nanoflower reduced the MRSA resistance gene mecR1 transcription, increasing the vulnerability of MRSA to ampicillin. Moreover, bacteria-specific aptamers were used to enhance the targeting of the nanoflowers to the MRSA cells. Naturally, some challenges need to be addressed when employing DNA nanotechnology. For example, nucleic acids must overcome barriers inside and outside the cell and resist degrading nuclease enzymes. In addition, the internal environment is complex. The lack of metal ions in target tissues directly affects the catalytic activity, and the effectiveness of DNAzymes depends on the assistance of the effective concentration of metal ions. When designing nanomaterials, metal ions are usually delivered simultaneously to ensure sufficient concentration [175].

#### Inorganic nanocarriers

Inorganic nanoparticles are mostly known within the antibacterial field to possess inherent antibacterial properties, with silver (AgNPs) being one of the most classic examples. However, this section is mainly devoted to using inorganic nanomaterials as drug carriers since reviews on inherently antibacterial nanoparticles are readily available in the literature. On the one hand, these can be conjugated to antimicrobial peptides for combination therapy and increased pathogen targeting ability [134]. Still, on the other hand, the cargo-carrying capacity of solid nanoparticles is relatively low and limited to the surface. Therefore, porous inorganic nanoparticles are more flexible candidates when aiming to formulate antibiotic drugs. For instance, mesoporous silica nanoparticles (MSNs) and metal–organic frameworks (MOFs) have high specific surface area and porosity, tuneable pore size, and controllable surface properties via ample functionalization strategies. Due to their robust inorganic matrix, they are especially suitable for delivering fragile bioactive molecules such as biomolecular drugs. Especially for MOFs, this biomineralization-based platform can even be used to provide mRNA or CRISPR/Cas9 to target resistance genes of microbes, which can generate a break inside the dsDNA of resistant bacteria, rendering them sensitive to antibiotics again [177].

Conversely, the pore size range of surfactant-templated MSNs typically lies within the size range of 3–12 nm, thereby excluding too-large biomolecular complexes from being sufficiently loaded within their pores. Thus, they have been typically utilized for the loading and delivery of small-molecule antibiotics [178–180]. The modular design prospects, moreover, allow for the adoption of co-delivery strategies in one system. For instance, in a recent study, Otri and co-workers co-delivered a linezolid gram-positive antibiotic that acts synergistically with gram-negative antimicrobial polymyxin B with one MSN system [181].

In this study, the linezolid was loaded into the MSN pores. At the same time, the polymyxin B was adsorbed onto the particle surface via electrostatic interactions, simultaneously functioning as a gatekeeper to block the premature release of linezolid from the pores. The MSN formulation was more effective against gram-negative *E. coli* and gram-positive *S. aureus* than the free drugs. Oliviera *et al.* constructed a gold@mesoporous silica (Au@MSN) core@shell material co-loaded with amoxicillin and ofloxacin and tested this nanocomposite against *S. aureus*, MRSA, *E. coli* and β-lactam resistant *P. aeruginosa* that in the best case even resulted in a complete circumvention of the resistance of MRSA. This example further showcases the vast design possibilities with this exceptionally flexible platform, allowing for combining more than one antibacterial mechanism into one system by using design elements (metals or other metal oxides) with inherently antibacterial properties together with antibiotic drugs [182–185]. Due to their robust nature, they are easily further formulated into dosage forms and different biomaterials [186–190].

### Enhancing the Efficacy, Bioavailability, and Targeting of Antibiotics

Similarly to within oncology, scientists are currently investigating passive and active targeting methods to research antibacterial treatment using nanomaterials. First, to give readers a clear overview of the mechanism of action of nanocarriers used in active and passive targeting, we briefly describe how they interact with bacterial membranes and how the drug is released to exert its effect. The interaction of NPs with bacterial cell membranes varies based on their characteristics, including surface charge, size, shape, or a combination of these properties. The interaction of nanoparticles with proteins, polysaccharides, and membrane lipid structures compromises bacterial membrane structure and integrity, resulting in membrane breakdown and cell death.

Metal NPs exhibit potent bactericidal activity by influencing bacterial viability via many mechanisms, including compromising membrane integrity by fusion and damage, inhibiting drug efflux pumps, obstructing electron transport, and denaturing proteins [191]. Cell envelope proves to be highly sensitive to metal ions and metal NPs. The cell envelope also acts as the outermost layer of the cell’s surrounding environment and shows critical importance in cell survival because of the electron transport channel. These changes include the ability of silver NPs to interact with the bacterial cell wall through charge attraction, allowing the particles to penetrate through the cell membrane. Furthermore, elevated levels of reactive oxygen species (ROS) that provoke oxidative stress and lipid peroxidation affect the fluidity, stability, and integrity of membranes, resulting in cell death following the interaction with NPs [192].

Furthermore, the behavior of nanoparticles with bacterial cells diminishes the ATP levels of the cells [193, 194]. Bacterial cells majorly produce ATP by glycolysis and respiration, with several nutrients being used for energy purposes [195]. ATP is released into the extracellular environment during development phases and is crucial for signaling and interaction among bacteria. A study investigating the effect of metal-NPs on bacterial cells showed a positive correlation between membrane permeability and increased cytoplasmic leakage, reduced total ATP levels and ATPase activity [193].

Modifiable lipid compounds such as liposomes are capable of membrane fusion, enabling drug transport across membrane barriers and offering great potential to combat antimicrobial resistance. In membrane fusion, the liposomes interact with the membrane through negative curvature lipids that facilitate the lipids mixing between the outer layers and cause the formation of stalks and hemifusion intermediates. Upon collapse of these intermediates, a fusion pore is formed, resulting in the compartment mixing and enhancing the delivery of contents [196].

Natural polymeric NPs such as chitosan, due to the presence of a cationic amine group, can electrostatically interact with bacterial negatively charged membrane lipid structure and increase the permeability by destabilizing phospholipids in the membrane. For instance, Dai *et al.* [197] demonstrated that polystyrene NPs functionalized by amine groups (PS-NH2) efficiently interact with cell membranes compared to PS-COOH (negatively charged particles). This result shows that the amine group has higher electrostatic interactions with bacterial membranes and facilitates the internalization of NPs through membrane penetration.

Hydrophobic interactions play a crucial role in bacterial membrane interactions since the enhanced hydrophobic characteristics of polymers can increase the attraction of NPs to the membrane surface. Therefore, considering the balance between the hydrophobic and hydrophilic regions in the polymer is crucial for NP performance in antibiotic delivery. An increase in hydrophobicity attracts a more significant amount of proteins, influencing the formation of the protein corona and altering their biological interactions. These properties affect the stability and targeting efficiency of the NPs in a biological environment and can either hamper or improve the delivery of therapeutics to bacterial cells [198].

#### Passively targeted delivery of antibiotics

Scientists are investigating passive and active targeting methods to research antibacterial treatment using nanomaterials. Passive targeting involves using nanomaterials to combat bacterial infections by either transporting antibacterial drugs or possessing inherent antibacterial properties such as silver nanoparticles (AgNPs), which disrupt bacterial cell membranes. Passive targeting enhances the efficiency of antibiotics while decreasing possible side effects. This can be achieved by increasing the levels of antibiotics around the bacterial cell environment [135, 199]. This phenomenon occurs by utilizing the enhanced permeability and retention (EPR) effect, a well-known concept from oncology. In infectious disease treatment, the EPR effect may likewise result in NP accumulation at the infection area due to improved vascular permeability and reduced lymphatic function in inflamed tissues [200]. Inflammation causes higher permeability, allowing NPs of sizes ranging from 10 to 200 nm to infiltrate and concentrate in these areas. This targeting strategy thus depends on altered physiological conditions in infected regions rather than ligand-receptor interactions (active targeting) [201, 202].

Şen Karaman *et al.* developed a core–shell nanocomposite (CeO2@mSiO2) using mesoporous silica as a nanocarrier platform. The nanocomposite contained capsaicin, an antimicrobial substance, and was covered with chitosan. The nanocomposite successfully halted the growth of pathogenic *E. coli* at a concentration of 50 μg/ml, and bacterial cells did not grow after a 4-h treatment. Experiments on *Drosophila melanogaster* larvae fed with bacteria demonstrated an enhanced suppression of bacterial growth in the gut compared to free capsaicin. Significantly, the nanocomposite exhibited no harm to mammalian cells, revealing its non-toxic nature [183].

AgNPs were investigated as a treatment for multi-drug-resistant *Salmonella* by Farouk *et al.* According to the findings, multidrug-resistant *Salmonella* showed susceptibility to AgNPs with MICs varying between ≤ 0.02 to 0.313 μg/mL (average of 0.085 ± 0.126 μg/mL) and MBCs varying between 0.078 to 1.250 μg/mL (average of 0.508 ± 0.315 μg/mL). Experiments conducted in both laboratory and living organisms verified the effectiveness of AgNPs in treating drug-resistant strains of *Salmonella*, with no negative impacts observed. The ability of AgNPs to eradicate bacteria is attributed to the interaction between the charges found on the bacterial cell wall and the metal ions. Moreover, the bactericidal properties are influenced by the stability, size, surface area, and shape of AgNPs. The Fourier-transform infrared spectroscopy (FTIR) analysis shows the strength of the created AgNPs by illustrating both the reduction of silver ions and the capping with polyvinylpyrrolidone [203].

Toti *et al.* investigated the application of biodegradable PLGA nanoparticles for delivering antibiotics to manage intracellular infections caused by *Chlamydia trachomatis* and *Chlamydia pneumoniae*. The MIC50 was roughly 40 ng/ml for azithromycin and around 5 ng/ml for rifampicin. The effectiveness of free drugs and nanoparticles was similar when administered immediately after infection. On the contrary, when administered 24- or 48 h post-infection, the accessible medicines showed limited effectiveness, while the encapsulated nanoparticles demonstrated better activity. This study has shown that PLGA nanoparticles can exploit lipid raft-mediated pathways for cellular entry, often in a non-specific manner [204].

#### Actively targeted delivery of antibiotics

Active targeting is described as utilizing biomimetic surface properties or biomolecular surface ligands that can selectively identify and attach to specific bacteria [205]. Antibodies, aptamers, and peptides are ligands that could improve the interactions between antibacterial agents and bacterial cells. These ligands show a high attraction for biomolecules or receptors that are expressed on bacterial cells or infected tissues. Through active targeting, the aim is to achieve a high level of specificity, binding affinity, and improved selectivity of antibacterial agents, thereby enhancing their inhibitory activities [206].

For instance, Manivasagan *et al.* developed antibody-conjugated and streptomycin-chitosan oligosaccharide-modified gold nanoshells (anti-STR-CO-GNSs) for synergistic chemo-photothermal therapy of drug-resistant bacterial infections. The fabricated nanoshells exhibited high photothermal conversion efficiency and released antibiotics in response to NIR and pH stimuli. *In vitro* experiments showed enhanced antibacterial effects against drug-resistant *Listeria monocytogenes* (LM) when treated with anti-STR-CO-GNSs and laser irradiation. After treating LM cells with CO-GNSs, STR-COGNSs, and anti-STR-CO-GNSs for 24 h, the amount of gold (Au) present in the cell membrane was measured.

The Au levels in the anti-STR-CO-GNSs were notably elevated compared to those in CO-GNSs and STR-CO-GNSs, suggesting that antibodies led to improved targeting capabilities. The anti-STR-CO-GNSs can attach to the cell membrane and eliminate the exteriors of bacterial cells, while STR can penetrate the cells and result in bacterial mortality. *In vivo*, results demonstrated that mice treated with anti-STR-CO-GNSs and laser irradiation exhibited the fastest recovery rate compared to the other groups within 7 days [205, 207].

Similarly, Calderona *et al.* investigated the efficacy of antibody-functionalized PLGA nanoparticles as a delivery system for rifampicin (Rif) against *S. aureus* and *E. coli*. Biotinylated polyclonal antibody against *S. aureus* binds via avidin–biotin complex on the surface of Rif-loaded PLGA nanoparticles developed through nanoprecipitation. The results showed that the antibody-coated nanoparticles exclusively targeted *S. aureus* in planktonic cultures and eradicated the bacteria at a 0.2 μg/ml concentration. In contrast, the non-targeted nanoparticles reduced bacterial proliferation by 105 compared to free Rif. In the biofilm model, nanoparticles, which were modified with antibodies, successfully decreased the presence of *S. aureus* within human macrophages, resulting in a 102 decrease in bacterial growth at a concentration of 0.5 μg/m compared to nanoparticles not modified by antibodies. Moreover, the nanoparticles with antibodies showed a better ability to prevent *S. aureus* biofilm formation than nanoparticles without antibodies [208].

Correspondingly, aptamer-conjugated nanomaterials employ short DNA or RNA molecules to target ligands [209]. Ucak *et al.* (2020) employed *S. aureus*-specific aptamers 2020 to modify PLGA NPs to deliver teicoplanin. The MIC values decreased from 1 µg/ml to 0.031 µg/ml for oxacillin-susceptible *S. aureus* strains via employing encapsulated teicoplanin in aptamer-PLGA nanoparticles. In clinical MRSA strains, the MIC decreased from 8 µg/ml when using regular teicoplanin to 0.125 µg/ml when using teicoplanin-aptamer-PLGA nanoparticles [210].

Peptide-conjugated nanomaterials employ short amino acid chains that can attach to bacterial cells [211]. To tackle intracellular *S. aureus* infections, which result in chronic diseases that are challenging to treat with conventional antibiotics, Huo *et al.* designed and synthesized TAT-KR-12 peptide combining elements from TAT peptide and KR-12 to enhance the cellular entry. The TAT segment acted like a"Trojan horse,"assisting in delivering the KR-12 peptide into target cells. Combining TAT with the KR-12 peptide allows for deliberate penetration of cell membranes, essential for interacting with and targeting intracellular bacteria. The results confirmed that TAT-KR-12 can be effectively used for treating different strains, such as clinical *S. aureus* (MIC: 16 μg/mL), MRSA (MIC: 16 μg/mL), *S. epidermidis* (MIC: 8 μg/mL) as well as eliminate *S. aureus* cells in both planktonic and biofilm forms [212].

A new method of delivering antibiotics via utilizing magnetic nanoparticles (MNPs) linked with a cell-penetrating peptide (CPP) was designed by Zhang and colleagues. They fabricated silica-coated iron oxide nanoparticles using a co-deposition technique. Then, they applied a polyvinyl alcohol (PVA) polymeric network through physical and chemical bonding and trapping with vancomycin (VCM). Next, the surface of the MNP was linked with a hexapeptide sequence, Gly-Ala-Phe-Pro-His-Arg, fabricated through solid phase synthesis to help NPs enter bacteria cells by interacting with the cell membrane. The involvement of particular amino acids, like arginine, within the CCP is crucial in the targeting procedure as the positively charged guanidine group in arginine can bind to negatively charged components of bacteria cell membranes, helping in the entry and uptake of NPs [213, 214]. Confocal microscopy showed NPs being taken up by both *S. aureus* and *E. coli* bacterial cells. Significant benefits of this system involve a quick, targeted drug distribution method, decreased drug amount, and equal efficacy against both Gram-positive and Gram-negative bacteria [215].

#### Nanotechnology-based treatment of biofilms

Developing effective treatments against biofilm-associated infections, especially those susceptible to developing antibiotic resistance is another current focus of extensive research. New techniques have been used for antibiotic delivery and formulation improvement to address these complications. Nanotechnology-based solutions have also demonstrated relatively vast potential in increasing the effectiveness of antibiotics in combating biofilm formation with negligible impact on eukaryotic cells in Fig. 3 (Biofilm formation) [216, 217].

Biofilms are clusters of different types of bacteria wrapped in a self-produced extracellular matrix that can bind to various surfaces and play an important role in antibiotic resistance. This protects the bacterial environment from the host immune system and prevents antibiotic drugs from penetrating into the matrix. This capability makes combating the infection more complicated, causing chronic infections. Therefore, dealing with biofilm-associated infections often necessitates higher antibiotic doses and extended treatment periods leading to increased adverse effects. Accordingly, studying and understanding the interaction of antibiotics, antibiotic NPs, or NPs loaded with antibiotic drugs with biofilms is a significant milestone toward effectively suppressing biofilm-associated infections [218].

Analyzing the interaction between NPs and biofilm can enable the design of targeted nano-antibiotics that improve the antibacterial activity and disturb or inhibit biofilm formation. This also contributes to diminishing the opportunities for developing resistance, as biofilms are well-known areas where antibiotic resistance mechanisms are cultivated due to the close positioning of bacteria in a shielded environment. As biofilms are connected to numerous persistent and difficult-to-eradicate infections in clinical environments, studying these interactions is vital for treatment advancement, leading to better clinical outcomes [219, 220]. Surface plasmon resonance (SPR) is a cutting-edge technology that allows real-time label-free monitoring of such NP-biofilm interactions. SPR records changes in refractive index near the sensor surface, which allows a detailed evaluation of the binding processes of NPs and their interactions with biofilms or bacterial environments [221].

Recently, multiparametric SPR was used to quantify the growth kinetics of *S. aureus* biofilms and their interaction with cerium oxide composite nanoparticles coated with a mesoporous silica shell (CeO2@MSNs), modified with different surface functionalizations leading to different net surface charges. The technique could show how various charges of the NPs generated different SPR responses due to interaction with the biofilm. Additionally, it was observed that positively charged polyethyleneimine-coated CeO2@MSNs exhibited better penetration and more robust interaction with the biofilm matrix than near-neutral acetylated CeO2@MSNs and negatively charged succinylated CeO2@MSNs. Thus, these observations support the capacity of SPR to promote NP-based antibacterial strategies. The SPR technique can be valuable in combating antibiotic-resistant infections by providing real-time information on the penetration and binding of NPs within biofilms [222].

## Clinical Progress and Challenges

### Current Drug Development Efforts for Combating Multi-Drug-Resistant Bacteria

The permanently troubling prospect of antimicrobial resistance has led researchers to put much effort into discovering and developing new antibiotics. Especially many academic groups are industriously contributing to the design and discovery of novel antibacterial agents using both natural sources [223–229] and synthetic approaches [230–234], as well as innovating novel therapeutic strategies such as employing adjuvants [235–237], photosensitizers [238] and multifunctional materials [239, 240] in addition to those discussed in this review. Furthermore, with the increasing computational power and available structural and biological data (e.g. ‘big data’ from ‘omics’ technologies), computer-aided drug design (including artificial intelligence, AI) is now routinely employed to facilitate the discovery and design of promising candidate compounds [241–246]. However, it will still take quite a while before all the most potential innovations get from the bench to the bedside, not the least due to the various limitations and challenges when translating preclinical research findings into the clinic [229, 247, 248]. In this chapter, we discuss the current clinical progress and translational challenges in the drug development against the MDR bacteria.

Table V contains a few potent compounds that have already reached the market or are at the advanced stage of clinical trials and exhibit great effectiveness against drug-resistant bacteria. Cefiderocol, a siderophore cephalosporin from a unique catabolic prodrug class, uses a Trojan horse strategy for the penetration of the outer membrane of Gram-negative bacteria [249]. The phase III study proved the efficiency of cefiderocol in common urinary tract infections with the resistance to carbapenem caused by Enterobacteriaceae and *P. aeruginosa* [250]. Omadacycline, an aminomethylcycline that is also the first of its kind, can get around the most common tetracycline resistance mechanisms while still active against Gram-positive bacteria, including MRSA [140]. The phase III clinical experiments verified omadacycline’s therapeutic efficacy in treating acute bacterial skin infections and community-acquired pneumonia (CAP) being non-inferior to linezolid [251].

**Table V:** Examples of Recently Developed Antibiotics Targeting Multidrug-resistant Bacteria

Antibiotic (market status)	Mechanism of Action	Targeted Bacteria	Clinical trial outcomes	Reference
Cefiderocol (FDA-approved 2019)	Siderophore cephalosporin	Carbapenem-resistant Enterobacteriaceae, *P. aeruginosa*, *Acinetobacter baumannii*	Demonstrated non-inferiority to high-dose meropenem for treating CUTI and demonstrated efficacy in treating infections caused by Gram-negative bacteria	[249, 250,]
Omadacycline (FDA-approved 2018)	Aminomethylcycline	MRSA, *Enterococcus faecalis*, *S. pneumoniae*	Non-inferiority to linezolid for treating ABSSSI and efficacy in treating CABP	[140]
Plazomicin (FDA-approved 2018)	Aminoglycoside	Carbapenem-resistant Enterobacteriaceae, *P. aeruginosa*	Non-inferiority to meropenem for treating CUTI and efficacy in treating bloodstream infections caused by CRE	[217]
Delafloxacin (FDA-approved 2017)	Fluoroquinolone	MRSA, *S. pneumoniae*	Non-inferiority to vancomycin/aztreonam for treating ABSSSI and efficacy in treating CABP	[220, 221]
Zoliflodacin (Phase III trials)	Spiropyrimi-dinetrione	*Neisseria gonorrhoeae*	Demonstrated efficacy in treating uncomplicated urogenital gonorrhea caused by *N. gonorrhoeae*, including strains resistant to fluoroquinolones	[250, 251]
Surotomycin (Phase III)	Cyclic lipopeptide	*Clostridioides difficile*	Demonstrated non-inferiority to vancomycin for treating CDI, particularly in achieving sustained clinical response and reducing recurrence rates	[252]

EMA – European Medicines Agency; FDA – US Food and Drug Administration; MRSA—methicillin-resistant *Staphylococcus aureus*; CUTI—complicated urinary tract infections; ABSSSI—acute bacterial skin and skin structure infections; CABP—community-acquired bacterial pneumonia; CRE—carbapenem-resistant Enterobacteriaceae; CDI—*Clostridioides difficile* infection

The next-gen aminoglycoside, plazomicin, showed high stability against the most common aminoglycoside-modifying enzymes produced by the carbapenem-resistant Enterobacteriaceae and *P. aeruginosa* [252]. A Phase III trial of a combination of meropenem and a beta-lactamase inhibitor, vaborbactam, documented an improved response compared to the best available therapy among patients with a CRE infection (including bloodstream infections that are often fatal conditions) [253].

Delafloxacine, an exceptionally non-zwitterionic fluoroquinolone, targets DNA gyrase and topoisomerase IV. If the molecules directly interact with the mutant sites, these two enzymes may exhibit resistance-causing mutations that affect the drug activity [254, 255]. Data collected during Phase III trials demonstrated that delafloxacin treatment is equivalent to vancomycin/aztreonam in acute bacterial skin and skin structure infections [256, 257]. In addition, delafloxacin is an effective treatment for community-acquired pneumonia [258].

Zoliflodacin has overcome the challenge of being the first member of a new class of antibiotics for treating *Neisseria gonorrhoeae* (*N. gonorrhoeae*) in over 30 years. This unique class of drugs, which prevents DNA gyrase from functioning by a mechanism similar to fluoroquinolones, is the first of its kind [259]. A Phase II trial demonstrated zoliflodacin's high efficacy in curing uncomplicated gonorrhea, including some resistant strains to fluoroquinolones [260]. Surotomycin, a ceftriaxone-type antibiotic composed of a cyclic lipopeptide, has proven effective by binding to the *C. difficile* spores and causing the pathogen to lose its viability. In Phase II investigations, ridinilazole was non-inferior to vancomycin for treating *C. difficile* infections and preventing recurrent infections [261].

In addition to the above success stories, several ongoing clinical trials investigate other potential candidates as novel antibiotics against drug-resistant pathogens. According to the most recent WHO report, “2023 Antibacterial agents in clinical and preclinical development: an overview and analysis” (data collected up to the end of 2023), there are nearly a hundred new chemical entities in the trials, out of which 26 are labeled as innovative depending on if they meet one or more of the four WHO criteria (new chemical class, new target, new mode of action or absence of cross-resistance) [59]. Over 60 trials target the WHO priority pathogens, including conventional antibiotics and non-traditional products (e.g., bacteriophages or antibodies).

The candidate agents extend current antibiotics classes with, for example, second-generation polymyxins (MRX-8 and upleganan, both in Phase I), an aminoglycoside (apramycin, Phase I), a tetracycline (zifanocycline, Phase I) and a macrolide (nafithromycin, Phase II) as well as new beta-lactam/beta-lactamase inhibitor (BL/BLI) combinations (ceftibuten/ledaborbactam, Phase I; aztreonam/nacubactam, Phase III; cefepime/taniborbactam, preregistration; cefepime/zidebactam, Phase III). In addition, the first-in-class compounds targeting novel bacterial targets, such as afabicin (Phase II), block fatty acid synthesis in *Staphylococcus* spp by inhibiting FabI, an enoyl-acyl carrier protein reductase [262] and murepavadin that inhibits an outer membrane protein (lipopolysaccharide transport protein, LptD of *P. aeruginosa*) are under investigation [263].

Despite all these advancements and promising new treatments, the WHO report states,"Overall, the clinical pipeline and recently approved antibiotics are insufficient to tackle the challenge of increasing emergence and spread of antimicrobial resistance.” Thus, persistent research is necessary to enhance available therapeutic options against the rising MDR infection risk. More recent antibiotics outlined in Table V serve as a testament to the ongoing research in the field of antimicrobial resistance and the movement away from the previous focus on specific bacterial pathogens to a more advanced clinical development program, unique therapeutic platforms, creative drug discovery strategies and solid late-stage testing methodologies [18, 264].

### Challenges and Limitations of Translating Preclinical Research

**Fig. 7:**
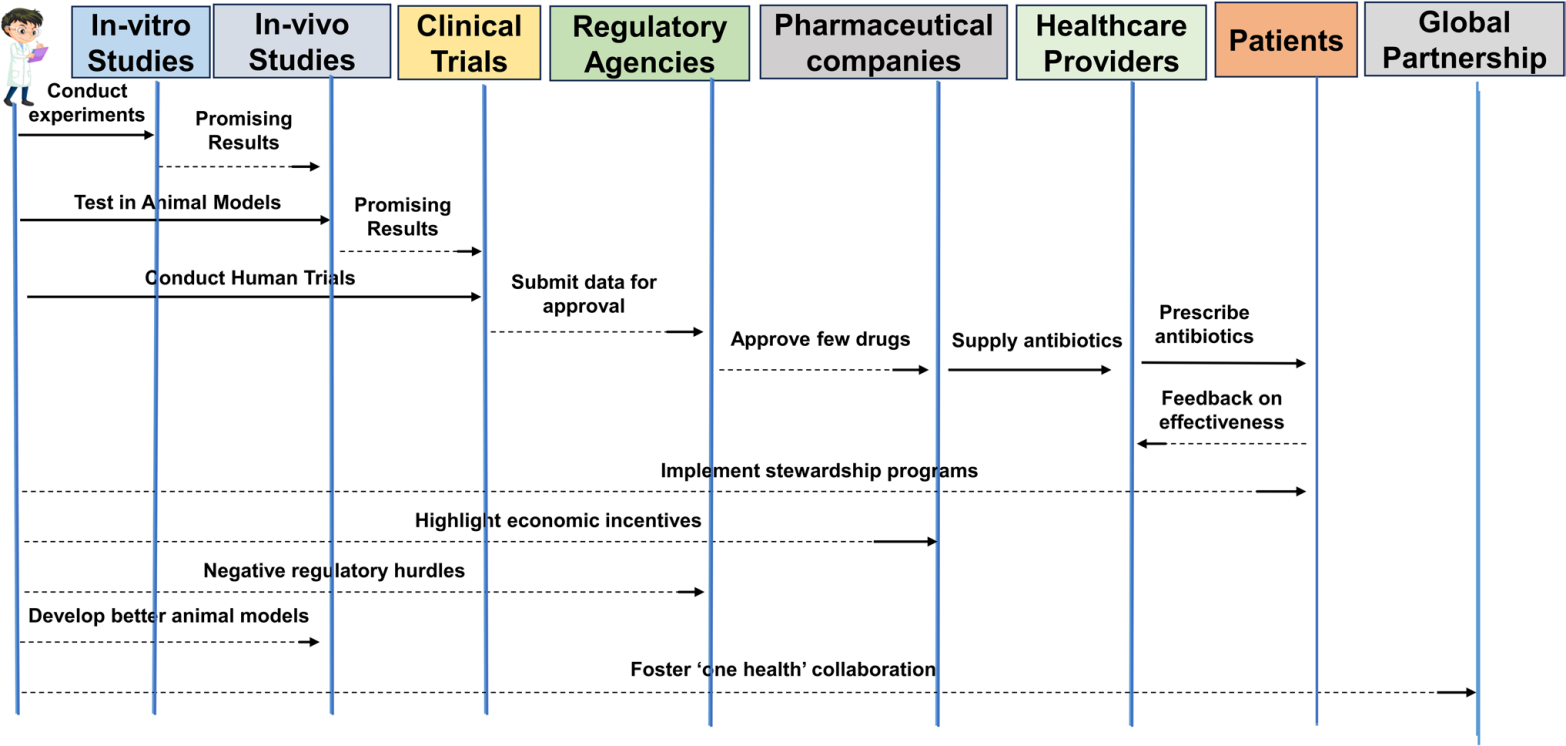
Challenges and limitations of translating preclinical research to the clinic. The straight arrows show direct actions or steps being taken by one entity and communicated to another, such as conducting experiments or testing in animal models. The dotted arrows represent likely responses, feedback, or more indirect actions, such as highlighting economic incentives or feedback on the effectiveness of prescribed antibiotics

Figure 7 illustrates the main challenges in translating the preclinical results from antibiotic resistance-related research into clinical use [58]. One of the significant challenges can be proving clinical effectiveness in humans by relying on *in vitro* data and animal model data, which may not always accurately mirror human disease progression and pharmacological response mechanisms. Subsequent research work in human subjects may be required to collect additional data on safety, tolerability, pharmacokinetic properties and efficacy in the target group of patients before a compound or drug can be implemented clinically [265, 266]. Some promising compounds based on *in vitro* and *in vivo* testing fail during human clinical trials due to the species-specific differences in pharmacokinetics and toxicology [267].

Therefore, more predictive animal models that better resemble human diseases are needed. Developing such models is a challenging task because of the complex physiology of humans [268, 269]. Another hindrance in this regard is the regulatory practices regarding the marketing authorization process [270]. The drug must go through three phases of clinical trials to establish its safety and effectiveness and be given regulatory approval. This is expensive, as well as time-consuming. On average, only one out of 5000 to 10000 medications would get approved after significant investment by pharmaceutical companies [271]. Hence, the current antibiotic developmental pipeline is inadequate [272]. The return on investment of pharmaceutical companies for new antibiotics is too low compared to the more profitable drugs for chronic diseases [273]. Subsidizing R&D costs through the government’s push-and-pull incentives may facilitate renewing the innovation for antibiotics.

One of the challenging elements in successful antibiotic stewardship is the consistency between laboratory and practical applications. The excessive and inappropriate use of antibiotics in medical practice and livestock farming is the basis for resistance [274]. Reducing the over/misprescription of antibiotics requires twofold action – behavioral change in healthcare providers and patients using stewardship programs and better, rapid tests that can discriminate bacterial and viral infections [275]. Naturally, the acceptance of new diagnostic techniques depends on their clinical usefulness and cost-effectiveness. Bovine antibiotic use for agricultural purposes must be carefully monitored through evidence of the transmission of antibiotic-resistant bacteria from animals to humans. Policymakers are responsible for striking a balance between increasing food production and potential economic impingements arising from the restrictions on antibiotic growth promoters.

There are at least five significant roadblocks in transforming preclinical results into clinically applicable antibiotics: absence of reliable animal models, high costs as well as low success rates in clinical trials, lack of economic incentives among producers, poorly chosen areas of research, and gaps in implementation of the best antibiotic stewardship practices [276]. Solving these hurdles requires a ‘one health’ global partnership involving the medical, industry, academic, government, agricultural and healthcare sectors. A broader global partnership could facilitate the development of new antibiotics from discovery to delivery.

## Conclusion

The development of antibiotic resistance in bacterial pathogens is particularly alarming and is one of the biggest challenges to healthcare facilities worldwide. Due to the general mechanisms such as plasmid exchange and selection pressure, bacteria resist the commonly used antibiotics, elevating the need to develop new antibiotics and modes of using them. These include, for example, searching for new bacterial targets (such as bacterial virulence factors), using microbial symbionts-derived antibacterial agents, and drug repositioning. The persistent phenomenon of antimicrobial resistance calls for inventing wholly different strategies to fight MDROs. Moreover, improved drug delivery systems, for example, employing nanotechnology, can enhance the effectiveness of antibiotics and decrease their acute toxicity. Several new-generation antibiotics are under development for clinical trials, including drugs with good prospects for overcoming the resistance problem if they pass through the development and approval processes. There are tremendous challenges when transitioning other novel agents from bench-to-bedside, such as the issue of showing efficacy for regulatory approval when resistance develops so fast. It is critical to strike a balance to ensure strict adherence to antibiotic stewardship principles while ensuring that more liberal regulatory review pathways are established to make it easier to roll out new agents. Thus, when scientific revelation, industrial innovation, regulatory oversight, and the healthcare system can collectively collaborate, there is a chance to adapt to the never-ending bacterial evolution and resistance.

## Data Availability

Data applicability statement is not applicable as this is a review article that is based on existing literature and we have not collected any data for this purpose.

## Declarations

### Use of Artificial Intelligence

During the preparation of this manuscript, Gemini AI (2024), chatGPT-4o and ZeroGPT were used for the purpose of improving the language in chapters ‘Antivirulence Approach’ and ‘Advancements in Antibiotic Formulation for Improved Delivery’. The authors have reviewed and edited the output and take full responsibility for the content of this publication.
